# Targeting FOSB with a cationic antimicrobial peptide, TP4, for treatment of triple-negative breast cancer

**DOI:** 10.18632/oncotarget.9612

**Published:** 2016-05-26

**Authors:** Chen-Hung Ting, Yi-Chun Chen, Chang-Jer Wu, Jyh-Yih Chen

**Affiliations:** ^1^ Marine Research Station, Institute of Cellular and Organismic Biology, Academia Sinica, Jiaushi, Ilan 262, Taiwan; ^2^ Department of Food Science, National Taiwan Ocean University, Keelung 202, Taiwan

**Keywords:** TP4, cationic antimicrobial peptide, triple-negative breast cancer, calcium, FOSB

## Abstract

Triple-negative breast cancer (TNBC) currently lacks a suitable therapeutic candidate and is thus difficult to treat. Here, we report that a cationic antimicrobial peptide (CAP), tilapia piscidin 4 (TP4), which was derived from Nile tilapia (*Oreochromis niloticus*), is selectively toxic to TNBC. TP4 acts by inducing an AP-1 protein called FOSB, the expression of which is negatively associated with the pathological grade of TNBC. We show that TP4 is bound to the mitochondria where it disrupts calcium homeostasis and activates FOSB. FOSB overexpression results in TNBC cell death, whereas inhibition of calcium signaling eliminates FOSB induction and blocks TP4-induced TNBC cell death. Both TP4 and anthracyclines strongly induced FOSB, particularly in TNBC, indicating that FOSB may be suitable as a biomarker of drug responses. This study thus provides a novel therapeutic approach toward TNBC through FOSB induction.

## INTRODUCTION

Breast cancer (BC) is the most common malignancy that causes death in women. Global gene-expression profile studies have classified breast cancers into different subtypes [1–3]. The subtypes which lack expression of estrogen receptors (ER), progesterone receptors (PR), and human epidermal growth factor receptor 2 (HER2) are clustered as triple negative BC (TNBC:ER^−^/PR^−^/HER2^−^). Hormone or targeted therapies are not usually effective against TNBC, with the exception of anthracycline or taxane-based conventional chemotherapy [4, 5]. However, TNBC patients, even after treatment with chemotherapy, often present with distant metastases and have poor prognosis. The main cause of chemotherapeutic agent failure is the development of multidrug-resistant (MDR) cancer cells under standard regimens [6], and chemotherapy also causes adverse side-effects [7]. Use of non-cross-resistant drugs [8–11] or biological agents [12] in combination with chemotherapeutic drugs is a possible option for TNBC patients with metastases. However, even though such options improve the outcome, the prognosis of metastatic TNBC patients remains poor.

Metastatic cancer cells that respond poorly to treatment usually possess negatively-charged phosphatidylserine (PS) or anionic structures on their outer membrane, in contrast to healthy cells that are normally zwitterionic [13, 14]. This characteristic allows some selective cytotoxic agents, such as cationic antimicrobial peptides (CAPs), to attack cancers through electrostatic interactions [15–17]. Cationic antimicrobial peptides (CAPs) are evolutionarily conserved components of the innate immune system, integral for activity against pathogens [18, 19]. The defensive capabilities of CAPs arise from their structures, which allow them to penetrate anionic bacterial membrane [16, 20] or cancer cells with negatively-charged outer membrane [21–23]. Treatment of cancer cells with large amounts of CAPs leads to transient membrane lysis [14, 24–29]. However, low concentrations of CAPs can trigger apoptosis [30, 31] and/or necrosis of cancer cells [24, 32–34]. Mechanisms of cancer killing by CAP involve changes in calcium homeostasis and induction of activator protein-1 (AP-1) [14, 23, 31, 35]. Calcium signaling appears to be activated early on in response to CAP-induced stress, and enhances downstream AP-1 signaling [23]. AP-1 members form a dimer with proteins of the JUN proto-oncogene (c-JUN) family (c-JUN, JUNB, JUND) or FBJ murine osteosarcoma viral oncogene homolog (FOS) family (c-FOS, FOSB, FRA1/2). The dimer composition of AP-1 activates downstream gene expression in response to cellular stimuli or in different cellular contexts, as well as controlling cell fate decisions [36]. In TNBC, FRA1 controls tumor cell growth and metastasis through repression of CDH1 in poorly differentiated cells [37, 38] which lack FOSB expression [39, 40]. However, little is known about the role of FOSB in TNBC.

TNBC cells with negatively-charged PS may be good candidate for CAP-based therapy. In the present study, we investigate the therapeutic potential of a CAP, tilapia piscidin 4 (TP4), derived from Nile Tilapia (*Oreochromis niloticus*) [41] for treatment of TNBC. Transcriptome analyses of TNBC cells and normal fibroblasts were carried out to identify potential molecular targets of TP4. The mechanism of action was investigated using *in vitro* BC cell models. The therapeutic efficacy of TP4 was further evaluated through intratumoral injection of null mice bearing BC xenotransplants. In addition, zebrafish embryonic models have been shown to have metastatic potential in BC xenografts, and the optical transparency of this model organism enables the study of tumor growth and metastasis [42]. Here, we employed zebrafish as a second model for testing the therapeutic potential of TP4 *in vivo*.

## RESULTS

### TP4 induces selective necrosis of TNBC cells

**Figure 1:**
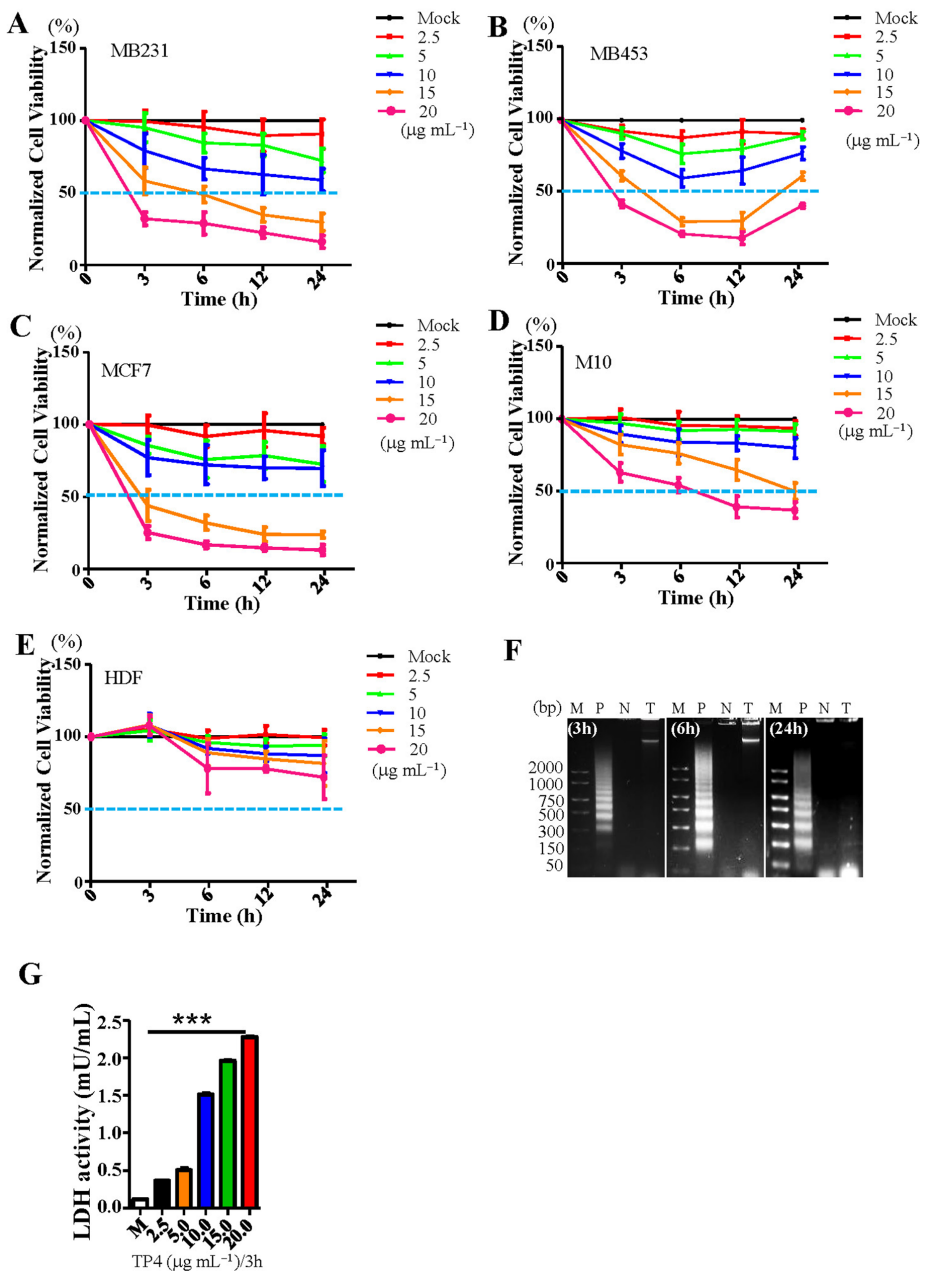
TP4 selectively kills breast cancer cells through inducing necrosis Cell viability in **A.** MB231, **B.** MB453, **C.** MCF7, **D.** M10, and **E.** HDF was determined by MTS assay following treatment with varying doses of TP4 (2.5-20 μg mL^−1^) at the indicated time-points (3-24h). Sextuplicate wells were analyzed for each assay. Results represent the mean±SD (*N* = 3, statistical analyses are shown in Supplementary Table S1). **F.** Detection of DNA fragmentation in TP4-treated MB231 cells by 2% agarose gel electrophoresis. Lane P: Positive control (Actinomycin D-treated HL60 cell lysate); lane N: Negative control (MB231 cell lysate); lane T: TP4-treated MB231 cell lysate; Lane M: DNA molecular weight marker. **G.** LDH levels in MB231 cells were determined following treatment with varying doses of TP4 (2.5-20 μg mL^−1^) at 3h. Sextuplicate wells were analyzed for each assay. Results represent the mean±SEM (*N* = 3, One way *ANOVA*: ***, *P* < 0.001 versus control, ns: not significant).

Different molecular subtypes of BC cell-lines (MDA-MB231, MDA-MB453, and MCF7) were subjected to the MTS assay to investigate whether TP4 can selectively kill BC cells *in vitro*. It was observed that treatment with 15 μg mL^−1^, 5.03 μM of TP4 is sufficient to kill over 50% BC cells at 6h, while the same dose had only minor effects on the viability of control normal human mammary epithelial cells (M10) or dermal fibroblasts (HDFs) (Figure 1A–1E, statistical analyses are shown in Supplementary Table S1). Genomic DNA samples from TP4-treated MDA-MB231 cells (denoted as MB231) were taken at different time-points and subjected to a DNA laddering assay; no obvious DNA fragmentation was observed after TP4 treatment, indicating that TP4 does not induce apoptosis in TNBC cells (Figure 1F). In addition, TUNEL staining of TP4-treated MB231 or HDF cells revealed very limited DNA fragmentation (Supplementary Figure S1A and S1B) and no obvious caspase3 activation was observed in TP4-treated TNBC cells (Supplementary Figure S1C and S1D). On the other hand, a necrotic marker, Lactate dehydrogenase (LDH), was significantly increased at 3h post-TP4 treatment in TNBC cells (Figure 1G). Taken together, these findings indicate that TP4 induces necrotic death in TNBC cells.

### FOS family members were induced by TP4 in TNBC cells

**Figure 2:**
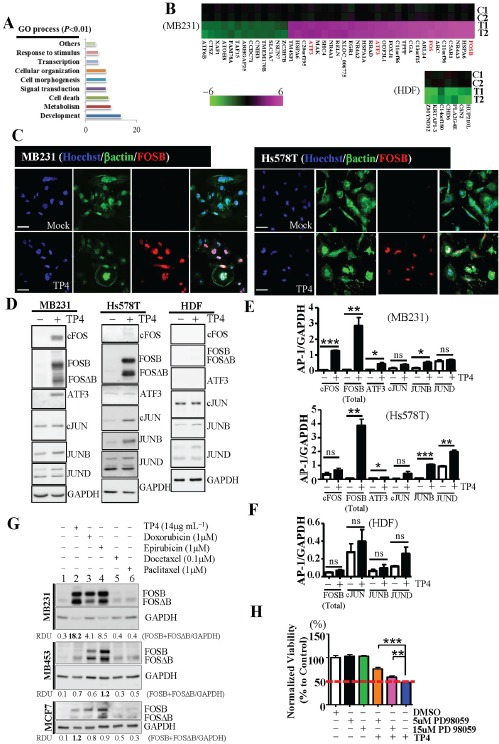
Induction of FOSB by TP4 in breast cancer cells **A.** Gene ontology (GO) analyses of the microarray study classified dysregulated genes into nine defined categories (*P* < 0.01). The graph shows the number of genes of each category that were found to be differentially expressed in MB231 cells following TP4 treatment, as compared to untreated controls. Annotation terms were determined using David 6.7 software. **B.** Heat maps depicting the changes of expression of genes in MB231 or HDF cells following TP4 treatment (scale bar indicates log_2_-fold changes). AP-1 transcription factor members are shown in red. C1, C2 and T1, T2 indicate the mock and TP4-treated samples collected from two independent assays, respectively. **C.** TP4- (14 μg mL^−1^) or mock-treated cells were stained with FOSB antibody (red) and βactin (green). Hoechst 33342 dye was used for nuclear staining (blue). Bar: 50 μm. **D.** Total lysates from MB231, Hs578T, or HDF cells with (+) or without (−) TP4 treatment were analyzed by Western blot using antibodies against GAPDH and FOS/JUN family proteins. **E**, **F.** Quantitative analysis of the blots shown in (D) using GAPDH as a control for normalization. Results represent the mean±SEM (*N* = 3, Student's *t*-test: *, *P* < 0.05; **, *P* < 0.01; and ***, *P* < 0.001. versus control, ns: not significant). **G.** Total lysates from control cells (lane 1), and cells treated with TP4 (lane 2), doxorubicin (lane 3), epirubicin (lane 4), docetaxel (lane 5), or paclitaxel (lane 6) were analyzed by Western blot using antibodies against GAPDH and FOSB. The relative amounts of FOSB plus FOSΔB in each lane are expressed as relative densitometric units (RDUs), calculated by dividing the FOSB plus FOSΔB signal by the GAPDH signal. **H.** MTS assay was used to measure cell viability in cells treated with PD98059 and TP4. Sextuplicate wells were analyzed for each assay. Results represent the mean±SEM (*N* = 3). Statistical comparisons of the differences between groups treated with or without PD98059 were performed using Student's *t*-test. ns: not significant; *, *P* < 0.05; ***, *P* < 0.001.

To characterize the downstream events which contribute to TP4-induced TNBC death, we analyzed gene expression profiles through microarray studies. Gene ontology (GO) analysis revealed that TP4 treatment caused dramatic changes in the gene expression profiles of TNBC cells (Figure 2A and 2B), but minor changes in HDF cells (Figure 2B). Of note, FOS members (FOSB, c-FOS) and ATF3 were significantly induced in TNBC cells (Figure 2B). Immunocytochemical studies and Western blotting confirmed that FOS members, particularly FOSB and FOSΔB (a truncated splice variant of FOSB), were induced in tested BC cell-lines (Figures 2C-2E and Supplementary Figure S2A-S2D). Unlike JUNB and JUND, cJUN was not significantly influenced in TNBC cells (Figure 2D and 2E). Neither FOS nor JUN family members were significantly activated in control HDF cells (Figure 2D and 2F). To explore the therapeutic role of FOSB, we investigated whether FOSB induction could be observed in TNBC cells during treatment with anthracycline or taxane-based chemotherapeutic agents. Interestingly, anthracyclines (doxorubicin and epirubicin) (Figure 2G, lanes 3 and 4) induced strong FOSB expression, comparable to that induced by TP4 treatment of TNBC cells (Figure 2G, lane 2). Taxane-based agents (docetaxel and paclitaxel), however, induced FOSB in MDA-MB453 (denoted as MB453) and MCF7 cells, but not in MB231 cells (Figure 2G, lanes 5 and 6). These findings suggest that TP4 and anthracyclines act through a similar therapeutic pathway in TNBC cells. In addition, Kyoto Encyclopedia of Genes and Genomes (KEGG) analysis of the microarray data revealed a significant effect of TP4 treatment on MAPK signaling (Table 1); this signaling pathway is known to increase AP-1 activity [43], and we further examined the molecules involved by Western blotting. We observed that active forms of both JNK and p38 were significantly decreased by TP4 treatment in TNBC cells, but not in control HDF cells (Supplementary Figure S3A-C). Activation of ERK proteins had no significant effect (Supplementary Figure S3A and S3B), but inhibition of ERK activity by PD98059 disrupted TP4-induced TNBC cell death, as shown by MTS assay (Figure 2H); these findings suggest that ERK signaling is required for TP4-activated cell death.

**Table 1:** KEGG categories of pathways significantly influenced by TP4 treatment of MB231 cells

Description	Term	Count	*P*-Value
MAPK signaling pathway	hsa04010	14	8.46E-03
Adherens junction	hsa04520	6	3.25E-02
Circadian rhythm	hsa04710	3	3.52E-02
p53 signaling pathway	hsa04115	5	7.22E-02
Pathways in cancer	hsa05200	13	7.66E-02

### TP4 induces FOSB to trigger TNBC cell death

**Figure 3:**
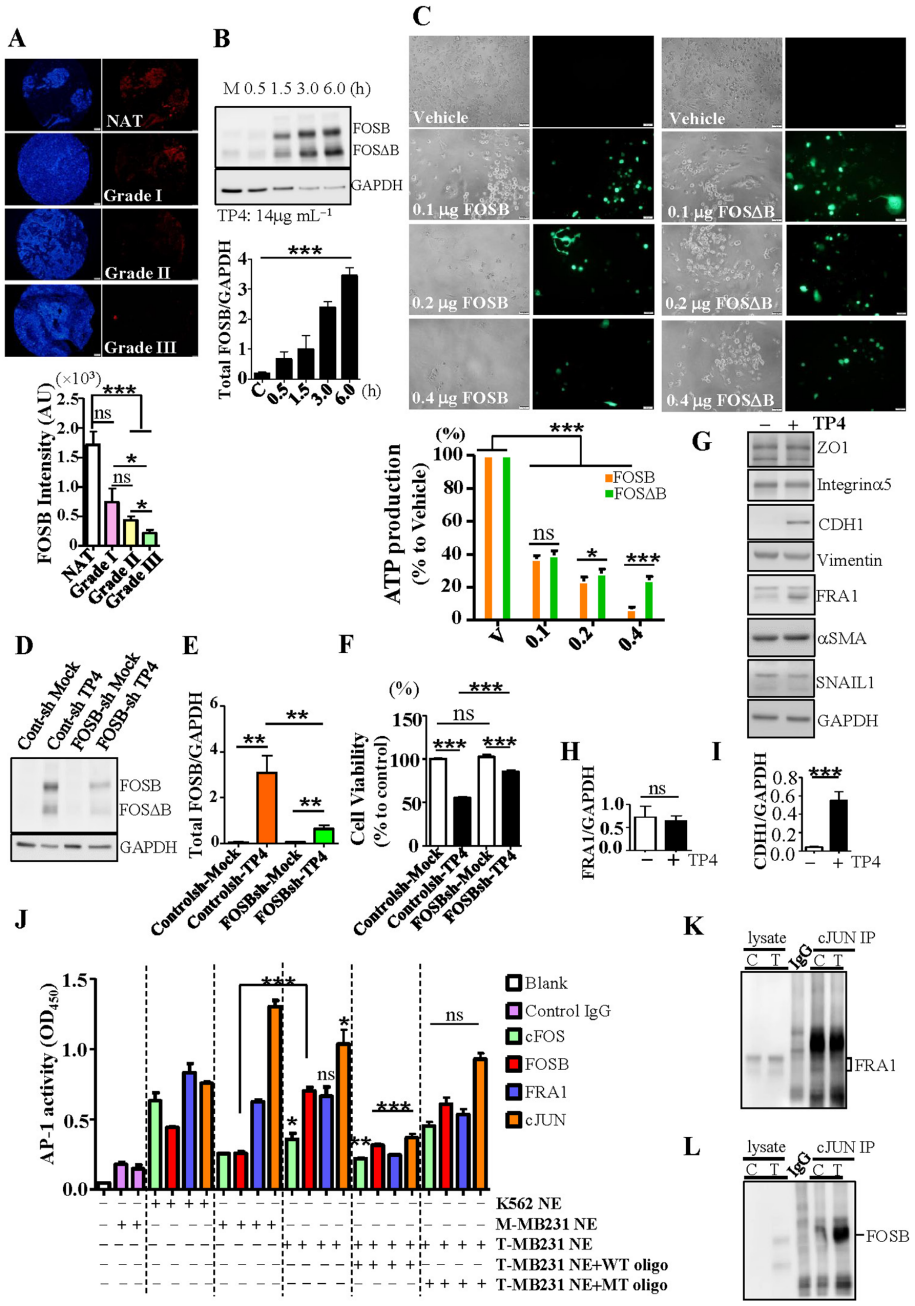
TP4 triggers TNBC cell death through FOSB induction **A.** Normal adjacent tissue (NAT, *N* = 26) and different grades of TNBC samples (*N* = 6, 19, 10 for grade I, II, III samples, respectively) were stained with FOSB (red) antibody and Hoechst 33342 (blue). Bar: 200 μm. Bottom graph, quantitation of the FOSB fluorescent signal indicated that FOSB level is associated with TNBC pathological grade. AU: arbitrary unit. **B.** Total lysates from mock (M) and TP4-treated groups were examined by Western blot. Bottom graph, quantitative analysis of total FOSB (FOSB plus FOSΔB) induction, normalized to GAPDH. **C.** Phase contrast and fluorescent images of MB231 cells transfected with FOSB or FOSDB vector. Bar: 50 μm. Cell viability was determined by ATP assay. At least fourteen replicate wells were analyzed for each dose. **D.** Total lysates from mock and TP4-treated (14 μg mL^−1^, 6h) MB231 cells transduced with control or FOSB shRNA lentivirus were analyzed by Western blot. **E.** Induction of FOSB levels, as normalized to GAPDH. **F.** The effect of TP4 treatment on the viability of the indicated cells, as determined by MTS assay. Sextuplicate wells were analyzed for each assay. **G.** Total lysates from MB231 cells (mock (−) or TP4-treated (+)) were analyzed by Western blot. **H**, **I.** Quantitative analyses of FRA1 (H) and CDH1 (I) levels, normalized to GAPDH. **J.** DNA-protein complexes were immunoprecipitated from mock (M-) or TP4-treated (T-) MB231 nuclear extracts (NEs) using the indicated antibodies. Forty picomoles of wild-type (WT) or mutated (MT) AP-1-binding oligonucleotides were used in the competition assay. K-562 cell NEs stimulated with TPA were used as a positive control. **K**, **L.** cJUN was immunoprecipitated from mock (C) or TP4-treated (T) NE with anti-cJUN antibody. Total lysates from mock or TP4-treated groups were used as positive controls. Immunoprecipitation with nonspecific IgG was performed as a negative control. Coimmunoprecipitation of FRA1 (K) and FOSB (L) with cJUN were examined by Western blot. Results represent mean±SEM (*N* = 3) by Student's *t*-test (A, D, E, H-J), one-way *ANOVA* (B), or two-way *ANOVA* (C). *, *P* < 0.05; **, *P* < 0.01; ***, *P* < 0.001, ns: not significant.

Strong induction of FOSB by TP4 in TNBC cells suggested possible involvement of FOSB in TP4-activated TNBC cell death. A previous study indicated that FOSB is highly expressed in normal ductal mammary epithelium, but not in poorly differentiated ductal carcinoma [40]. To address whether FOSB expression is associated with TNBC progression, we analyzed FOSB expression in various grades of tumor samples from TNBC patients by immunohistochemical analysis. Expression of FOSB in breast normal adjacent tissue (NAT, *N* = 26) was found to be stronger than expression in grade II (*N* = 19) and grade III (*N* = 10) TNBC samples (Figure 3A, *P* < 0.001). Grade I samples (*N* = 6) showed a trend towards decrease, but were not statistically different to NAT (Figure 3A). These results indicate that FOSB expression is decreased during TNBC progression, and suggest that FOSB may be detrimental to TNBC development. We then evaluated whether the induction of FOSB by TP4 is associated with TNBC death. As demonstrated by Western blotting, the increase of FOSB in TNBC cells treated with TP4 is time-dependent (Figure 3B) and is correlated with the timing of TP4 induced-cell death (Figure 1A). Transient expression of FOSB or FOSΔB (0.1-0.4 μg) in TNBC cells resulted in substantial cell death as compared to the vehicle control, as determined by ATP assay (Figure 3C, *P* < 0.001). Interestingly, TNBC cells were more resistant to FOSΔB expression than FOSB expression, at high concentration (Figure 3C, *P* < 0.001). We proceeded to examine whether FOSB knock down disrupts TP4-activated TNBC cell death. FOSB-knockdown MB231 cells were generated through transduction with lentiviral particles containing 4 specific shRNA constructs (19-25 nucleotides, including the hairpin). Our Western blotting data indicate that TP4 treatment caused significant FOSB induction in control cells (*P* < 0.01), but not FOSB-knockdown cells (Figure 3D and 3E). The results acquired from MTS assay showed that FOSB knockdown significantly protected MB231 cells against TP4-induced death (Figure 3F). We next investigated whether the molecular composition of AP-1 complexes are influenced by strong induction of FOSB in TNBC cells. It was previously shown that FRA1 is associated with the epithelial-to-mesenchymal transition (EMT) as a key factor involved in TNBC progression [38]; however, the level of FRA1 was not influenced by TP4 treatment, as shown by immunoblotting (Figure 3G and 3H). Surprisingly, levels of CDH1 were significantly increased (Figure 3G and 3I), but those of other EMT-related proteins (ZO1, Integrin α5, Vimentin, αSMA, and SNAI1, Figure 3G) were unaffected. We proceeded to determine the activity of each FOS family member. AP-1 activation was quantified by incubating nuclear extracts from TNBC cells treated with or without TP4 with oligonucleotides containing a tetracycline response element (TRE); DNA-protein complexes were subsequently isolated using antibodies against c-FOS, FOSB, FRA1, and c-JUN. In the absence of TP4 (mock control), the signal-to-background ratios of c-FOS, FOSB, FRA1, and c-JUN activation (represented by OD_450_) were 1.4:1, 1.4:1, 3.5:1, and 8.8:1, respectively (Figure 3J). Cells treated with TP4 exhibited a 1.4 and 2.8 fold increase of c-FOS and FOSB activity, respectively, as compared to mock controls (*P* = 0.0291 and *P* < 0.001) (Figure 3J); such an increase was not observed for FRA1 (*P* = 0.5593, Figure 3J). Interestingly, c-JUN activity was decreased by TP4 treatment (*P* = 0.0272) (Figure 3J). Coimmunoprecipitation of cJUN confirmed an association between c-JUN and FRA1 (Figure 3K), and the cJUN-FOSB immunocomplex was identified after TP4 treatment of TNBC cells (Figure 3L). These results suggest that the induction of FOSB by TP4 in TNBC cells possibly alters AP-1 complex composition and thereby causes cell death.

### TP4 causes mitochondrial dysfunction

**Figure 4:**
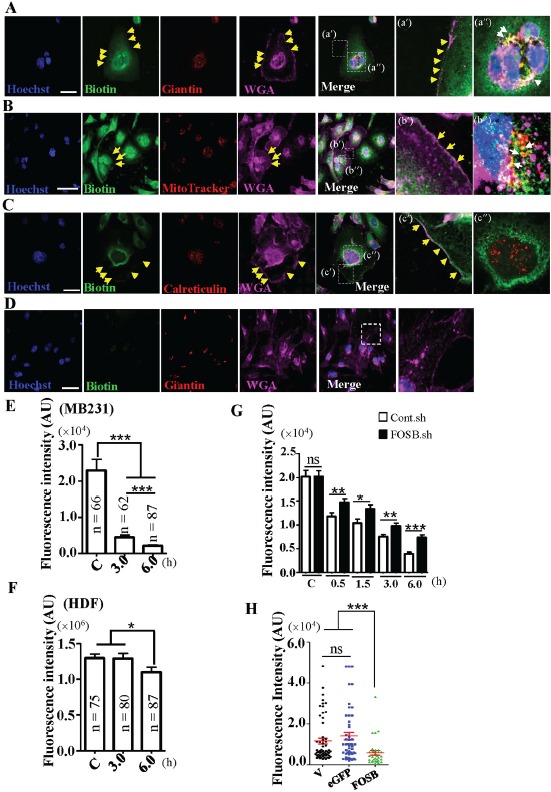
TP4 is bound to the TNBC cell membrane and intracellular organelles **A–D.** Cellular localization of biotinylated-TP4 in MB231 (A-C) and HDF cells (D). Cells were stained with biotin (green), Golgi marker (giantin; red) (A,D), ER marker (calreticulin; red) (B), and mitochondrial marker (mitotracker; red) (C) antibodies. The plasma membrane was labeled with Alexa Flour-647-conjugated WGA (purple). Hoechst 33342 was used for nuclei staining (blue). Boxed regions are shown magnified in the panels to the right of the merged images. Yellow and white arrows indicate co-localization of biotinylated-TP4 with plasma membrane and Golgi or mitochondria, respectively. Bar: 50 μm. **E**, **F.** Quantitation of the fluorescent signals, indicating that mitochondrial membrane potential was significantly decreased in TP4-treated MB231 cells (E). Statistical comparisons between mock and TP4-treated cells were performed using Student's *t*-test. ns: not significant; *, *P* < 0.05; ***, *P* < 0.001. **G.** Quantitation of the mitochondria fluorescent signals in Controlsh– and FOSBsh–MB231 cells treated with TP4 (14 μg mL^−1^ for 0.5−6.0 h), indicating that mitochondrion intensity could be partially restored in FOSB-knockdown MB231 cells by TP4 treatment. Statistical comparisons between mock and TP4 treatment groups were performed using Student's t-test (N = 50 in each group). ns: not significant; *, P = 0.0221; **, P < 0.01; ***, P < 0.001. AU: arbitrary unit. **H.** Quantitation of the fluorescent intensity in vehicle control (V), eGFP-transfected, and FOSB-transfected MB231 cells, indicating that mitochondrion fluorescent intensity was decreased in FOSB-transfected MB231 cells. Statistical comparisons between mock and TP4-treated groups were performed using Student's t-test. ns: not significant; ***, P < 0.001.

To characterize the mechanism of action of TP4 and the role of FOSB induction, we examined the cellular localization of TP4 in TNBC cells. Cells treated with biotinylated TP4 (14 μg mL^−1^) for 1h were co-stained with biotin, organelle-specific antibodies/dye (Calreticulin for the ER; Giantin for the Golgi; and MitoTracker for the mitochondria), and fluorescent dye-conjugated WGA (for the plasma membrane). TP4 was observed to be bound to the Golgi, mitochondria, and plasma membrane as evidenced by strong co-localization of the biotin signal with Giantin (Figure 4A, indicated by white arrows), MitoTracker (Figure 4B, indicated by white arrows), and WGA (Figure 4A–4C, indicated by yellow arrows), but not with the ER (Figure 4C). Importantly, only weak background staining against biotin was observed in the nuclei of the HDF control (Figure 4D), suggesting that normal cell membranes are unlikely to be recognized by TP4. The observation that TP4 is selectively bound to the mitochondria led us to examine whether TP4-activated BC toxicity is associated with mitochondrial dysfunction. Immunocytochemical staining through potential-dependent accumulation of MitoTracker revealed a significant loss of mitochondrial membrane potential in TNBC cells at 3 and 6h post-TP4 treatment as compared to the control group (*P* < 0.001) (Figure 4E and Supplementary Figure S4A), while no significant difference was observed for HDF cells (Figure 4F and Supplementary Figure S4B). We next investigated whether FOSB induction contributes to the loss of mitochondrial membrane potential in TNBC cells. FOSB knockdown partially prevented the loss of mitochondrial membrane potential in response to TP4 treatment as compared to the control cells (Figure 4G, Supplementary Figure S4C and S4D). Interestingly, FOSB-transfected cells showed a significant loss of mitochondrial membrane potential compared to un-transfected control or vector transfected control (Figure 4H and Supplementary Figure S4E-G). These findings suggest that TP4 induces a loss of mitochondrial membrane potential prior to FOSB induction; subsequent FOSB induction may further contribute to mitochondrial dysfunction.

### Mitochondrial calcium leakage caused by TP4 induces FOSB

**Figure 5:**
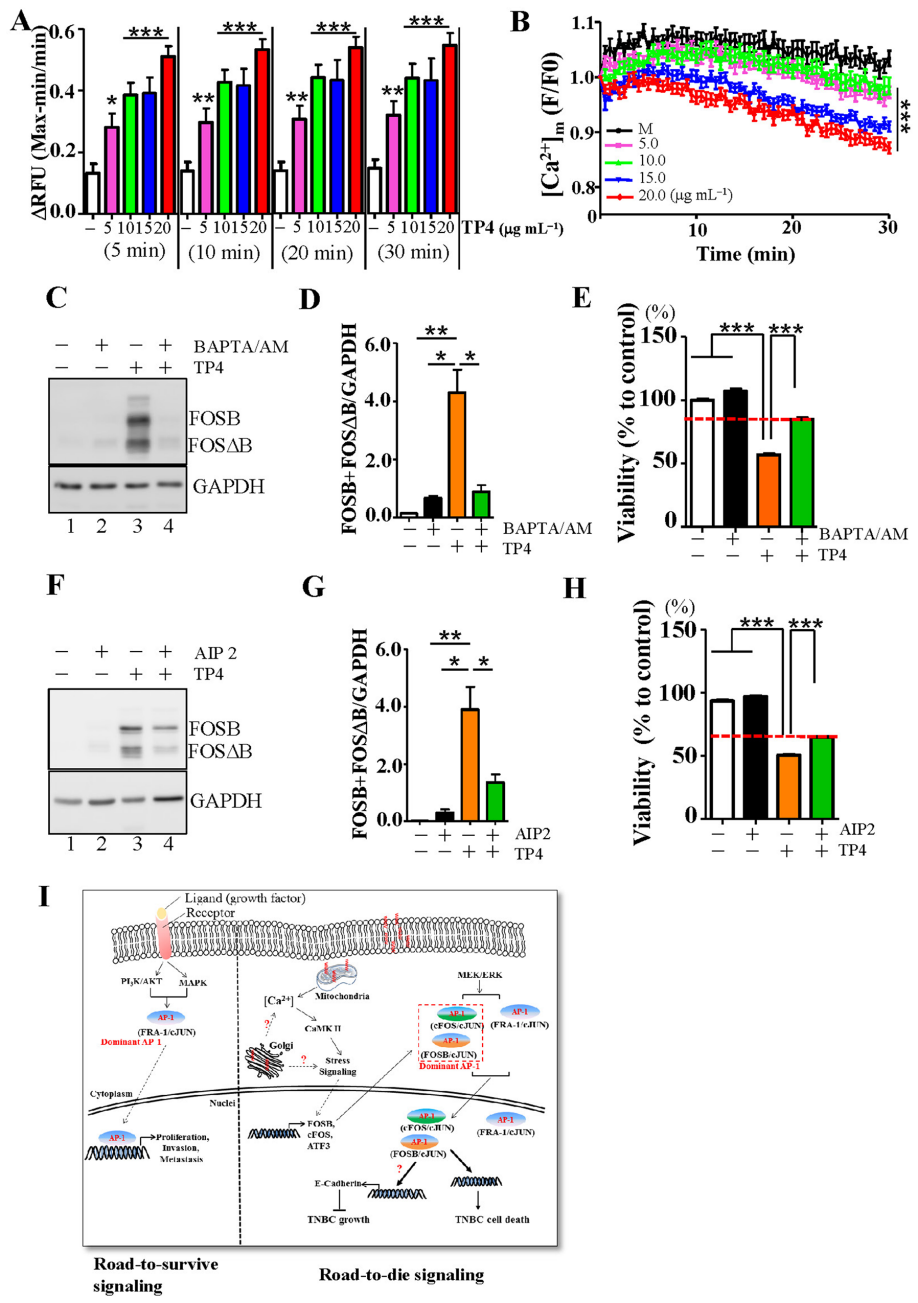
FOSB induction in TNBC cells requires calcium signaling **A.** Ca^2+^ levels were measured by the addition of fluorescent Ca^2+^ indicator (Fluo-4) after treatment with the indicated doses of TP4 for 5-30 min. Octuplicate wells were analyzed for each assay. Results represent the mean±SEM (*N* = 3, Student's *t*-test: *, *P* < 0.05; **, *P* < 0.01; ***, *P* < 0.001). **B.** Mitochondrial Ca^2+^ levels were measured kinetically using a fluorescent Ca^2+^ indicator (Rhod-2 AM) after treatment with the indicated doses of TP4 every 30 sec for 30 min. Results represent the mean±SEM (*N* = 3, one-way *ANOVA*: ***, *P* < 0.001). **C**, **F.** Total lysates from control (lane 1), BAPTA/AM (calcium chelator)-treated or AIP2 (CaMKII inhibitor)-treated cells (lane 2), TP4-treated cells (lane 3), and combination-treated cells (lane 4) were analyzed by Western blot, using antibodies against GAPDH and FOSB. **D**, **G.** Quantitative analyses of the blots shown in (C, F); levels of FOSB plus FOSΔB were normalized to GAPDH. Results represent the mean±SEM (*N* = 3, Student's *t*-test: *, *P* < 0.05; **, *P* < 0.01). **E**, **H.** Cell viability was measured in cells treated with Ca^2^^+^ chelator or CaMKII inhibitor and TP4. Sextuplicate wells were analyzed for each assay. Results represent the mean±SEM. Statistical comparisons of the differences between groups treated with or without inhibitors were performed using Student's *t*-test. ***, *P* < 0.001. **I.** Proposed mechanism-of-action of TP4 against TNBC. TP4 binds the cell membrane and selectively binds the mitochondria. This in turn results in Ca^2+^ release and induction of FOSB expression. FOSB/c-JUN becomes the predominant AP-1 complex that triggers downstream cell death.

In our earlier work, we showed that CAP induces AP-1 to trigger cancer cell death through calcium signaling [23]. We next examined whether Ca^2+^ homeostasis is influenced by TP4 treatment in TNBC cells. Intracellular Ca^2+^ levels were measured using fluo-4 Ca^2+^ indicators at 5-30 min after treatment of TNBC cells with TP4 (Figure 5A). A significant increase in the Ca^2+^ levels of cells treated with 5-20 μg mL^−1^ TP4 for 5 min as compared to the mock control (*P* < 0.001) was observed, indicating that TP4 treatment altered Ca^2+^ homeostasis in TNBC cells. However, it is likely that TP4 does not bind the ER (Figure 4C), the intracellular Ca^2+^ store, but instead binds the mitochondria (Figure 4A and 4B), which takes up Ca^2+^ released from the ER, suggesting that the increase of intracellular Ca^2+^ may be due to leakage from mitochondria. We tested this possibility by using a mitochondrial Ca^2+^ indicator, Rhod-2 AM, to dynamically monitor the Ca^2+^ level upon TP4 treatment. We observed that the Ca^2+^ levels in cells treated with 5-20 μg mL^−1^ TP4 for 30 min exhibited a trend towards decrease as compared to the mock control (*P* < 0.001), indicating that TP4 treatment disrupted Ca^2+^ dynamics in mitochondria (Figure 5B). In addition, we addressed whether TP4-induced, Ca^2+^-triggered stress responses cause downstream FOSB induction. Pre-treatment of TNBC cells with BAPTA-AM, a Ca^2+^ chelator, prior to TP4 treatment disrupted FOSB induction and TP4-activated TNBC cell death, as compared to the mock control (Figure 5C–5E). Moreover, application of AIP II, a calcium/calmodulin-dependent protein kinase (CaMK) II inhibitor, to block Ca^2+^-triggered downstream signaling resulted in a trend towards decrease, but not complete block, of FOSB induction compared to mock control (Figure 5F and 5G) and partly prevented TP4-induced TNBC cell death (Figure 5H). Overall, these results indicate that TP4 is bound to the mitochondria, disrupts Ca^2+^ homeostasis, and ultimately induces downstream FOSB to activate TNBC cell death (Figure 5I).

### TP4 inhibits tumor growth in a nude mouse xenograft model

**Figure 6:**
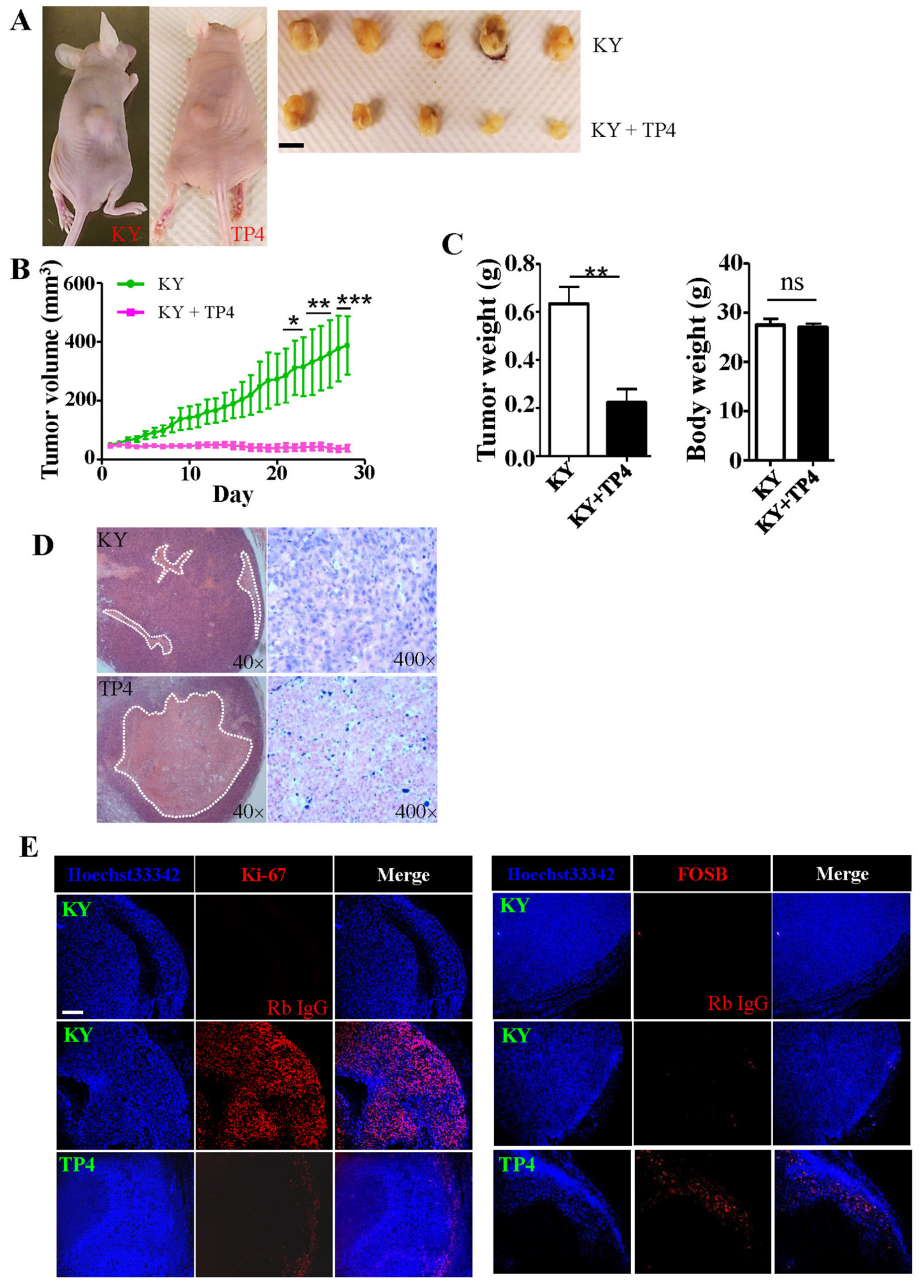
TP4 inhibits TNBC xenograft growth in nude mice **A.** Xenograft growth in nude mice (*N* = 5). **B.** Quantitation of tumor size at the indicated days after the commencement of TP4 treatment. Statistical comparisons between KY and TP4-treated groups were performed by two-way *ANOVA* with post hoc analysis (Bonferroni test). ns: not significant; *, *P* < 0.05; **, *P* < 0.01; ***, *P* < 0.001. **C.** Xenograft tumor weight (left) and mouse body weight (right) were determined when the mice were sacrificed (Student's *t*-test, **, *P* < 0.01; ns, not significant). **D.** H&E staining of xenograft tumors. White dotted lines mark necrotic regions. **E.** Immunohistochemical staining of Ki-67-positive (left) and FOSB-positive (right) cells in xenograft tumors. Bar: 200 μm.

To evaluate the effects of TP4 treatment on tumor growth *in vivo*, we subcutaneously transplanted TNBC cells into nude mice (*N* = 5), and assessed tumor growth daily for 28 days. A group of nude mice with xenografts were treated with TP4 every two days once the tumor reached a certain size. Significant differences in tumor growth between control (KY jelly alone) and TP4 (KY jelly plus TP4)-treated groups were observed (*P* < 0.001) (Figure 6A and 6B). TP4-treated tumors grew into smaller tumor masses than those of control groups (*P* = 0.0017) (Figure 6C, left), but no significant differences in body weight were observed between each group of mice (Figure 6C, right). Control experiments showed that KY jelly (10 μL plus 50 μL distilled water) was well absorbed after injection into age-matched control nude mice (Supplementary Figure S5A and S5B). Pathological studies confirmed that a large portion of the central region is necrotic in intratumoral TP4-treated groups (Figure 6D). A dramatic decrease in cells positive for the proliferation marker Ki-67 was observed in tumor tissue sections from TP4-treated groups, paralleling the macroscopic findings (Figure 6E, left). In addition, FOSB expression was also detected within the tumor (Figure 6E, right). Collectively, these results indicate that TP4 kills TNBC cells *in vivo*.

### TP4 prolongs the survival of TNBC xenograft zebrafish

**Figure 7:**
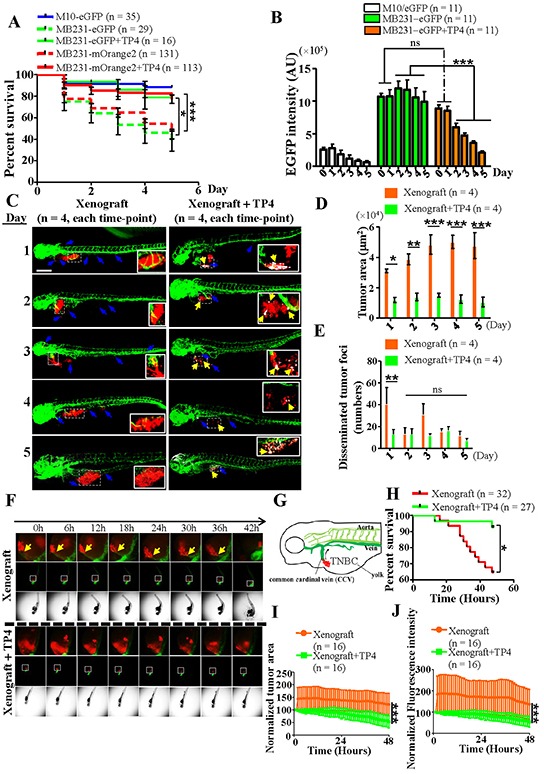
TP4 treatment prolongs survival in TNBC xenograft zebrafish **A.** Survival analysis of M10 and TNBC cells with or without TP4 treatment. Statistical comparisons were performed by Log-rank test. *, *P* < 0.05; ***, *P* < 0.001. **B.** Quantitation of the eGFP fluorescent signals in M10 and TNBC xenografts with or without TP4 treatment (3 μg mL^−1^ for 5d). Statistical comparisons between mock and TP4-treated cells were performed using Student's *t*-test (*N* = 11). ns: not significant; ***, *P* < 0.001. AU: arbitrary unit. **C.** Transgenic zebrafish (*fli*:*eGFP*) with mOrange2-expressing TNBC xenografts (red) underwent mock or TP4 treatment (3 μg mL^−1^ for 5d) and were then stained with FOSB antibody (white). Each panel is a merged image of photographs taken of the posterior and anterior parts. Boxed regions are shown magnified in the lower-right corner of the figures. Blue arrows indicate disseminated tumor foci. Yellow arrows indicate TNBC xenografts with positive FOSB signals. Bar: 200 μm. **D**, **E.** Quantitation of the primary tumor area (D) and disseminated tumor foci (E) in TNBC xenograft zebrafish. Results represent mean±SEM, and were analyzed by Student's *t*-test (*N* = 4 in each group). ns: not significant; *, *P* = 0.0221; **, *P* < 0.01; ***, *P* < 0.001. **F.** Time-lapse study of transgenic zebrafish (*fli*:*eGFP*) with mOrange2-expressing TNBC xenografts (red) during a single treatment with TP4 (3 μg mL^−1^). Time series images were taken every 1 h, including *z*-stacks. Selected planes within 48hrs are shown. Arrows in xenograft zebrafish indicate blood vessel invasion of TNBC cells. Boxed regions are shown magnified in the images above the figures. **G.** Schematic drawing of TNBC xenograft migration through the common cardinal vein (CCV) in zebrafish. **H.** Survival analysis of TNBC xenografts with or without TP4 (3 μg mL^−1^, single treatment) at 72-120 hpf. Statistical comparisons of survival curves between groups were performed by Log-rank test. *, *P* < 0.05. **I**, **J.** Quantitation analysis of xenograft tumor growth, based on normalized tumor area (I) and fluorescence intensity (J), in zebrafish with or without TP4 treatment. Results represent mean±SD, and were analyzed by two-way *ANOVA*; ***, *P* < 0.001.

To further investigate the therapeutic ability of TP4, we generated a TNBC xenograft zebrafish model with which to study the ability of TP4 to inhibit TNBC migration and invasion. A schematic indicating the treatment procedures and analytic approaches used in this study is shown in Supplementary Figure S6. Fluorescence reporter TNBC cell-lines were first obtained by transfection of M10 or TNBC cells with eGFP or mOrange2 expression vectors, followed by antibiotic selection (Supplementary Figure S7A). Survival analysis at 48 hours post-fertilization (hpf) revealed no obvious toxic effects of injection of non-tumorigenic eGFP-expressing M10 cells (800-1,200 cells per embryo, zebrafish survival rate > 90%, Figure 7A). In contrast, injection of eGFP- and mOrange2-expressing TNBC xenografts showed an unexpected increase in zebrafish embryo mortality at 168 hpf to about 38.3% and 44.8%, respectively (Figure 7A). Before evaluating the therapeutic activity of TP4, we examined TP4 toxicity in zebrafish. Serial dilutions of TP4 (0.125 ng mL^−1^-20 μg mL^−1^) were added to the zebrafish culture medium; we observed that TP4 doses of 1 and 2 μg mL^−1^ had no obvious toxic effects on normal zebrafish (Supplementary Figure 7B). However, further testing revealed poor therapeutic efficacy of these doses in eGFP-expressing TNBC xenograft zebrafish (Supplementary Figure S7C and S7D). As most wild-type zebrafish (> 75%) treated with 3 μg mL^−1^ (1.01 μM) TP4 were still viable at 168 hpf (Supplementary Figure S7B), we used this dose in subsequent experiments. TP4 (3 μg mL^−1^, administered daily) treatment significantly prolonged survival of eGFP- and mOrange2-TNBC xenograft zebrafish (78.9% and 82.5%) in comparison with mock-treated groups (*P* = 0.0149 and *P* < 0.0001, respectively) (Figure 7A). The therapeutic efficacy of TP4 in a single xenograft zebrafish was determined by quantitation of the eGFP fluorescent signal through days 0-5 (48-168 hpf). In control M10 xenograft zebrafish, the eGFP fluorescent signal exhibited a gradual trend towards decrease; however, such a trend was not observed in TNBC xenograft zebrafish (Figure 7B, left and center). In the TP4-treated groups, the eGFP fluorescent intensity was significantly decreased through days 2-5 in comparison with the mock control (*P* < 0.001), indicating a positive therapeutic effect of TP4 *in vivo* (Figure 7B). To address the mechanism underlying TP4 treatment, we performed whole-mount staining to determine whether TP4 treatment exerted any TNBC cell-autonomous effects. We report that TNBC cells in the TP4-treated group, but not the mock-treated group, presented with positive FOSB staining (Figure 7C). In addition, the xenograft tumor area (*P* < 0.05 compared to the non-treated group) and the numbers of disseminated tumor foci (*P* < 0.01 at day 1 compared to the non-treated group) were decreased upon TP4 treatment (Figure 7D and 7E). Together, these results indicate that TP4 may contribute to autonomous elimination of TNBC through FOSB induction. We also investigated whether any non-TNBC cell autonomous effects induced by TP4 benefit cancer cell elimination *in vivo*. To this end, the expression profiles of certain genes involved in innate immunity against some pathogen infections in zebrafish embryo were determined by qPCR [44]. We observed that application of TP4 (3 μg mL^−1^, administered daily) to wildtype zebrafish generally resulted in a significant decrease of immune gene expression over time (Supplementary Figure S8A-S8G, left), except at some specific time-points (*Il8* increased at Day 1 and *Il10*/*Ifnφ1* increased at day 5); however, TP4 treatment had no significant effects on *Tnf*α expression (Supplementary Figure S8E, left) as compared with the un-treated control. In TNBC xenograft zebrafish embryos, TP4 treatment was observed to significantly enhance immune responsive gene expression at around day 2 (Supplementary Figure S8A-G, right) as compared to un-treated groups. These results suggest that TP4 may enhance innate immunity in TNBC xenograft zebrafish embryos. Furthermore, the finding that TNBC xenografts enhance mortality in zebrafish led us to further investigate the mechanisms involved through high-content imaging. Time-lapse imaging revealed that TNBC cells migrated and invaded developed blood vessels, causing substantial abdominal edema, curvature of the trunk, and death (Figure 7F, G, Supplementary Movies S1 and S2). TNBC xenograft zebrafish that received a single treatment of TP4 (3 μg mL^−1^) exhibited prolonged survival in comparison with the non-treated group (92.6% vs 65.6%, *P* < 0.05) (Figure 7H, Supplementary Movies S3 and S4), and also contained reduced quantities of TNBC, as revealed by a gradual decrease in xenograft TNBC tumor area (Figure 7I) and fluorescence intensity (Figure 7J). These findings indicate that TNBC metastasis and invasion are possibly a major cause of zebrafish death, and that TP4 treatment eliminates TNBC growth *in vivo*.

## DISCUSSION

In this study, we report that TP4 is selectively toxic to BC cells. *In vitro* and *in vivo* evidence indicate that TP4 may be suitable as a novel agent to treat TNBC. TP4 damages TNBC cells through the ERK/FOSB/cJUN axis controlled by Ca^2^^+^ signaling. Activation of FOSB in TNBC requires Ca^2^^+^, which is transduced by selective binding of TP4 to the mitochondria. In addition, induction of CDH1 by TP4 may also contribute to TNBC suppression. Widely-used anthracyclines also induced FOSB in TNBC cells. This finding, together with the observation that FOSB overexpression triggers TNBC cell death, indicates that FOSB may be a novel therapeutic candidate.

Alteration of AP-1 transcription factor activity is sufficient to promote tumorigenesis [36–38]. We have shown that the level of FOSB is significantly decreased in grade II/III TNBC samples (moderately differentiated or poorly differentiated tumor) (Figure 3A); this is in agreement with previous reports of strong FOSB expression in normal breast epithelia or well-differentiated breast tumor [39, 40]. These results suggest that a molecular switch of FOS family member expression (from c-FOS/FOSB to FRA1) occurs during TNBC progression. FRA1 has been shown to control the development of aggressive breast cancers, as well as TNBC [37, 38, 45–47]. High levels of FRA1 activate genes involved in EMT to trigger TNBC proliferation, invasion, and metastasis [38, 45]. We showed that the level of FRA1 is not altered by TP4 in TNBC. However, the tumor suppressor protein CDH1, which is known to be suppressed by FRA1-associated signaling, was induced by TP4 (Figure 3G and 3H). This finding suggests that the FRA1-driven EMT program may have been disrupted by FOSB-induced changes in the dominant AP-1 complex in TNBC cells (Figure 5I). We also observed that the activities of both JNKs and p38 MAPKs were suppressed by TP4 in TNBC cells, but not in HDF cells (Supplementary Figure S3A). JNKs and p38 both possess oncogenic or tumor-suppressive functions [48]. In TNBC, both kinases are required for tumor development and progression, as mediated through cJUN/AP-1-activated signaling pathways [38, 49, 50]. Here, the decrease of JNKs and p38 activities in MB231 cells may destabilize cJUN/FRA1 or other cJUN/AP-1 complexes, leading to the transcriptional inactivation of genes involved in TNBC cell progression. Interestingly, *FOSB* exhibits differential patterns of post-transcriptional regulation among different subtypes of BC. A greater proportion of full-length *FOSB* transcripts were found to be induced by TP4 or anthracyclines in MCF7 cells; conversely, less full-length but more *FOSΔB* transcripts were found in MB453 cells (Figure 2G). Since overexpression of high concentrations of FOSΔB is less toxic than FOSB overexpression to MB231 cells (Figure 3C), it is possible that the FOSB, and not FOSΔB, predominantly contributes to BC cell death. In support of this possibility, MB453 cell re-growth was observed at 12h post-TP4 treatment, while MCF7 and MB231 cells did not regrow (Figure 1A–1C).

Anthracyclines, but not taxane-based drugs, strongly induce FOSB in BCs; anthracyclines kill cancer cells through causing torsional stress and nucleosome destabilization [51, 52]. An earlier study showed that doxorubicin causes histone eviction and transcriptome alterations in cancer cells [53]; therefore, FOSB may be a suitable biomarker for the response to anthracyclines in BC cells. The mechanisms by which TP4 and anthracyclines induce FOSB and trigger BC cell death are different. While some BC-targeting peptides were reported to be localized to the nucleus and cause DNA fragmentation [14], we did not observe a strong nuclei staining pattern of TP4 in BC cells (Figure 4A–4D), suggesting that TP4 may not influence the transcriptome in BC cells through the same manner as anthracyclines. In addition, doxorubicin treatment increased the mitochondrial Ca^2^^+^ level in BC cells [54] and triggered apoptotic cell death [55]. TP4, however, caused Ca^2^^+^ leakage from mitochondria (Figure 5B), leading to necrosis (Figure 1E and 1G). Blockage of Ca^2^^+^ signaling with the calcium chelator eliminated FOSB induction and disrupted TP4-induced cell death (Figure 5C–5E); however, we did not examine whether FOSB induction by anthracyclines can be eliminated through Ca^2^^+^ blockage, and whether elimination of FOSB activation can disrupt anthracycline-induced BC cell death. Furthermore, we observed that the induction level of FOSB in TNBC cells is higher than that in MCF7 and MB453 cells. It is not known whether hormonal or HER2 receptor status in different subtypes of BC correlates with the efficacy of chemotherapy and the induction levels of biomarkers. Elucidation of cross-links between signaling pathways may facilitate greater understanding of drug resistance in different BC subtypes.

Intratumoral injection of TP4 caused extensive necrosis of TNBC in xenograft tumor (Figure 6D and 6E) without causing adverse side-effects (Figure 6C), suggesting that intratumoral injection of TP4 may be of practicable use for further therapeutic regimens. Another critical concern is that necrotic cell death caused by TP4 may trigger severe immunogenicity *in vivo* and further damage surrounding tissues. Non-apoptotic cell death is more immunogenic than apoptotic cell death, as it triggers heat shock protein (hsp) induction [56]. Analysis of the microarray results revealed significant induction of a series of *HSP* genes (refer to Supplementary Datasheet S1), suggesting that strong immunogenicity may be induced by TP4 *in vivo*, but may not be observed in assays using immunodeficient null mice. However, intratumoral injection of CAP has been proposed to exert dual actions against tumor xenografts through directly inducing tumor lysis and subsequent activation of immune responses [57]. Here, we observed that innate immunity was enhanced by TP4 treatment in both zebrafish embryos and TNBC xenograft embryos (Supplementary Figure S8). An enhancement of immune responsive gene expression was observed in TNBC xenograft embryos, particularly at days 1-3 (Supplementary Figure S8A-G, right). As TP4 did not appear to cause an overall induction of immune responses in normal embryos, we propose that the immunity may be enhanced by the self-defense mechanism against TNBC xenografts in zebrafish embryo. In addition, it is possible that amphiphilic TP4 can passively diffuse into embryos [42] and directly or indirectly cause TNBC death, which may trigger further immune responses. Our results support the hypothesis that non-TNBC cell autonomous effects in zebrafish embryo exist and help eliminate cancer cells; however, the key players involved (*e.g.* neutrophils and macrophages) and the signaling pathway(s) required for their activation still need to be identified.

In conclusion, we have identified (i) TP4 as a novel cytotoxic peptide possibly suitable for breast cancer therapy, and (ii) FOSB as a biomarker of the response to TP4 and anthracyclines, particularly in TNBC. In contrast to previous reports that TNBC can be suppressed through FRA1 signaling inhibition, we observed that TNBC cell growth can be disrupted by FOSB activation. Therefore, our findings are of importance for TNBC treatment through modulation of AP-1 levels. FOSB plays an opposing role to FRA1 in the regulation of TNBC fate decision.

## MATERIALS AND METHODS

### Reagents

TP4 (H-FIHHIIGGLFSAGKAIHRLIRRRRR-OH) and TP4 biotinylated at the N-terminus were synthesized and purified by GL Biochem Ltd. (Shanghai, China) as previously described [23, 41]. Autocamtide-2 related inhibitory peptide II (AIP II) and PD98059 were purchased from EMD Millipore. BAPTA-AM [1,2-Bis(2-aminophenoxy)ethane-N,N,N′,N′-tetraacetic acid tetrakis(acetoxymethyl ester)], Paclitaxel, Docetaxel, Epirubicin hydrochloride, and Doxorubicin hydrochloride were purchased from Sigma.

### Cell culture and stable clone selection

Cell-lines [MB231 (BCRC 60425), Hs578T (BCRC 60120), MB453 (BCRC 60429), MCF7 (BCRC 60429), M10 (BCRC 60197)] used in this study were purchased from the Bioresource Collection and Research Center (BCRC) and the standard cell culture procedures and conditions provided by the BCRC were followed. HDF cells were cultured as previously described [23]. With the exception of MB231 and MB453, all cells were cultured at 37°C with 5% CO_2_. For the cell viability and transfection assay, 1 × 10^4^ cells [5 × 10^3^ M10 cells were seeded and cultured for 48h to allow the cells sufficient time for attachment] were seeded into the wells of a 96-well plate and cultured overnight. For the transfection assays, cells were transfected with 0.1-0.4 μg FOSB/FOSΔB expression plasmid (Origene Technology Inc.) and cell viability was determined after 72h. The transfection efficiencies (number of cells expressing eGFP/all cells) of the MB231 transfection assays were determined by observing ten randomly selected fields (from three independent transfections) of control GFP plasmid transfections under an inverted microscope (Olympus, IX71) coupled to a digital camera (Olympus DP80), using an 10× objective lens (LCPlanFI 20× /0.40 Ph1). CellSens standard software (Olympus) was used for image acquisition. During the drug treatment assay, inhibitors (PD98059, BAPTA-AM, and AIP II) were added 30 min prior to TP4, and cell viability was determined at indicated time-points. Transfection was performed using LipofectAMINE™3000 (ThermoFisher Scientific), according to the manufacturer's recommendations. Knock-down cells were generated by transducing MB231 cells with pre-synthesized FOSB (or control) shRNA lentiviral particles (Santa Cruz Biotechnology), and selecting puromycin-resistant cells in accordance with the manufacturer's standard protocol. MB231 or M10 cells stably expressing eGFP or mOrange2 were generated through transfection with peGFP-puromycin or pmOrange2-C1 plasmid, followed by puromycin (5 μg mL^−1^) or G418 (500 μg mL^−1^) selection as described above.

### Cell viability assay

Cell viability was quantitatively analyzed using the CellTiter-Glo® Luminescent Cell Viability Assay kit (ATP assay) and CellTiter 96® AQueous Non-Radioactive Cell Proliferation Assay kit (MTS assay) (Promega) in accordance with the manufacturer's protocol. For MTS assay, 1 × 10^4^ cells were seeded into the wells of a 96-well plate and cultured overnight [5 × 10^3^ M10 cells were seeded and cultured for 48h to enable sufficient well attachment]. Cells were subsequently treated with different doses of TP4 (2.5-20 μg mL^−1^) and harvested at the indicated time-points (3-24h). Reaction mixtures (20 μL : MTS+PMS, using a ratio 20:1) were directly added to the cells, and the plates were incubated for 3h at 37°C. Absorbance at 490nm is directly proportional to the number of living cells in culture and was measured using a photometer (SpectraMax® i3, Molecular Devices). ATP assay was performed as previously described [23]. Lactate dehydrogenase (LDH) assays were performed by quantitatively measuring cell lysis with a Cytotoxicity Detection Kit^PLUS^ (LDH) (Roche) in accordance with the manufacturer's protocol. The LDH standard was purchased from Cayman Chemical. Briefly, 1 × 10^4^ cells were seeded into the wells of a 96-well plate and cultured overnight. Culture media were replaced with fresh medium containing 1% FBS and cells were subsequently treated with different doses of TP4 (2.5-20 μg mL^−1^). Supernatants were harvested at 3h. After centrifugation at 200× *g* for 5 min to remove cell debris, supernatants were collected and 50 μL were aliquoted from each well into a new microplate. Reaction mixtures were then added and incubated for 15 min at RT. Stop solution was added to the well, and absorbance at 490 nm was determined with a reference wavelength of 600 nm.

### DNA laddering assay

DNA fragmentation was analyzed using the Suicide-Track™ DNA Ladder Isolation Kit (EMD Millipore) in accordance with the manufacturer's standard procedures. Sufficient DNA samples from TP4 treatment groups were extracted by collecting cells from three (control) or seventeen (TP4-treated groups) 10 cm^2^ dishes. Precipitated DNA samples were analyzed by 1.5× agarose gel electrophoresis.

### Transcriptome analysis

Total RNA samples were extracted from MB231 and HDF cells treated with TP4 (14 μg mL^−1^) for 6h. Total RNA (0.2 μg) was amplified using a Low Input Quick-Amp Labeling kit (Agilent Technologies, USA), and the cDNA was labeled with Cy3 (CyDye, Agilent Technologies, USA) during the *in vitro* transcription process. Cy3-labled cRNA (0.6 μg) was fragmented to an average size of about 50-100 nucleotides by incubation with fragmentation buffer at 60°C for 30 min. Corresponding fragmented labeled cRNA was then pooled and hybridized to an Agilent SurePrint G3 Human V2 GE 8×60K Microarray (Agilent Technologies, USA) at 65°C for 17h. After washing and drying using a nitrogen gun blowing, microarrays were scanned with an Agilent microarray scanner at 535 nm to detect Cy3. Scanned images were analyzed using Feature extraction 10.5.1.1 software (Agilent Technologies, USA); image analysis and normalization software was used to quantify signal and background intensity for each feature.

### AP-1 transcription factor activation assay

Activation of AP-1 was determined using the TransAM AP-1 kit (Active Motif, Inc), as previously described [23].

### Coimmunoprecipitation and western blot

Nuclear extracts were prepared as previously described [23]. Equal amounts of nuclear extract (200 μg) were used for immunoprecipitation (IP) using Dynabeads protein G (ThermoFisher Scientific), in accordance with the recommended protocol. cJUN antibody (ThermoFisher Scientific, clone C.238.2) was used for immunoprecipitation. Total cell extract preparation and Western blot were performed as previously described [23]. Equal amounts of boiled lysate (20 μg of total cell extract) were separated on acrylamide gels, and then transferred to PVDF membranes. The membranes were incubated in blocking solution (0.1 M PBS, 5% non-fat milk, 0.2% Tween-20) for 1h at room temperature (RT), and then incubated in the same solution with primary or secondary antibodies (GE Healthcare Life Science). Primary antibodies were as follows: c-FOS (Cell signaling, 9F6, 1:1,000), FOSB/FOSΔB (Cell Signaling, 5G4, 1:1,000), FRA1 (Cell Signaling, D80B,1:1,000), ATF3 (EMD Millipore, 6B8, 1:500), JUNB (Cell Signaling, C37F9, 1:1,000), JUND (EMD Millipore, 1:1,000), c-JUN (EMD Millipore, 6A6.2, 1:2,000), Vimentin (Abcam, EPR3776, 1:5,000), CDH1 (Cell Signaling, 24E10, 1:1,000), Integrin α5 (Cell Signaling, 1:1,000), Glyceraldehyde-3-phosphate dehydrogenase (GAPDH, EMD Millipore, clone 6C5, 1:10,000), αActin (smooth muscle) (αSMA, OriGene Technologies, 1:5,000), SNAI1 (ABGENT, N-term D24, 1:500), and ZO1 (ThermoFisher Scientific, 1:1,000). Membranes were visualized with enhanced chemiluminescence (Immobilon Western Chemiluminescent HRP substrate, Merck Millipore) and detected by an imaging system (UVP, BioSpectrum™ 500). Signal intensities were determined by densitometric analysis (AlphaInnotech) using the AlphaImager program. The results were expressed as relative densitometric units (RDU) (the densitometric units of FOSB+FOSΔB divided by those of GAPDH).

### Calcium measurement

Calcium (Ca^2+^) levels were determined using the Fluo-4 Direct Ca^2+^ assay kit (ThermoFisher Scientific) and Rhod-2 calcium indicator (ThermoFisher Scientific), as recommended by the manufacturer. Briefly, 1 × 10^4^ cells were seeded into a well of a 96-well plate and cultured overnight. Eight replicates were performed for each condition. Cytosolic calcium was measured by adding 2× Fluo-4 Direct™ reagent (final probenecid concentration of 5 mM) directly to each well, and then incubating the plates for 30 min at 37°C, and subsequently for 30 min at RT. Cells were treated with TP4 (5-20 μg mL^−1^) for 5, 10, 20, or 30 min. Fluorescence was subsequently measured using a fluorescence reader (SpectraMax® i3, Molecular Devices), using instrument settings appropriate for excitation at 494 nm and emission at 516 nm. Ca^2^^+^ levels are presented as relative fluorescent units (ΔRFU), determined using the following equation: F-F_min_/F_min_,where F_min_ denotes the background-subtracted pre-stimulus fluorescence level. Mitochondrial Ca^2+^, was measured by incubating cells with 2 μM Rhod-2-AM and 0.02% pluronic F-127 for 30 min at 37°C. After three washes in D-PBS, cells were incubated for 30 min in culture medium at 37°C. Cells were treated with TP4 (5-20 μg mL^−1^) and fluorescence was determined kinetically every 30 sec for 30 min using a fluorescence reader with instrument settings appropriate for excitation at 552 nm and emission at 581 nm. Mitochondrial Ca^2^^+^ levels are presented as relative fluorescent units F/F0, where F0 denotes the un-stimulated fluorescence level.

### Immunocytochemical, immunohistochemical, and whole-mount studies

The plasma membrane and mitochondria were stained by pre-incubating biotinylated-TP4 treated cells (14 μg mL^−1^, 3h) with Alexa Flour 647 dye-conjugated wheat germ agglutinin (WGA) (5 μg mL^−1^) (ThermoFisher Scientific) for 10 min at 37°C or with MitoTracker® Red CMX-ROS probe (200 nM) (ThermoFisher Scientific) for 45 min at 37°C prior to cell fixation. Cells were then fixed with 4% PFA (in PBS) for 15 min, and permeabilized with 0.1% Triton X-100 in PBS (PBST) for 12 min at RT. After blocking with 5% BSA in PBST, the cells were incubated overnight at 4°C with Biotin (Santa Cruz Biotechnology, 39-15D9, 1:500), Calreticulin (Merck Millipore, 1:500), Giantin (Abcam, 1:1000), or FOSB (1:500) antibody. Cells were then washed three times with TBS-T (20 mM Tris–HCl, pH 7.4, 137 mM NaCl, and 0.1% Tween-20), and incubated for 1h at RT with secondary antibodies (1:500; ThermoFisher Scientific) conjugated to the appropriate fluorescent dye. Hochest33342 was used for nuclear staining. The fluorescent signal (which is proportional to functional mitochondria) was quantitatively determined using Image J software. Human breast adjacent normal tissue array (BRN801a) and TNBC tissue array (BR487a) were purchased from US Biomax, Inc. The TNBC tissue array included grade I (well-differentiated), grade II (moderately differentiated), and grade III (poorly differentiated) patient samples. Commercially-available human tissue samples were used in accordance with the regulations of the“Human Subject Research Ethics Committee” of Academia Sinica. Paraffin sections were immunostained with FOSB antibody (1:50) and Hochest 33342. Fluorescent images were obtained with an inverted microscope (Olympus, IX71) coupled to a digital camera (Olympus DP80), using a 4× (UPlanFI 4× /0.13 PhL) objective lens. CellSens standard software (Olympus) was used for image acquisition. The fluorescent FOSB signal was quantitatively determined using Image J software. For whole mount staining, xenograft zebrafish were fixed using 4% PFA for 1h at RT. After four washes for 5 min each in PBST (1% Triton-X-100), fish were incubated in blocking buffer (PBS+1% triton-X-100+10% FBS) for 1h at RT. Fish were then washed twice with blocking buffer and incubated with FOSB antibody (1:50) for 2 days in blocking buffer. After a further three washes for 1h each in PBST, fish were incubated with secondary antibody conjugated to Alexa Flour 647 for 2h at RT. Fish were then washed three times with PBST for 10 min each at RT. After mounting (tissues or cells) with fluorescent mounting medium (ProLong Gold Antifade Reagent, ThermoFisher Scientific), images were obtained with an FV1000 laser-scanning confocal microscope (Olympus), using a 10× (Olympus UPlanSApo 10×, N.A. 0.40) or 60× objective lens (Olympus UPlanSApo 60×, N.A. 1.35, oil). ASW2.1 software (Olympus) was used for image acquisition, disseminated tumor foci quantitation, and the measurement of primary tumor area.

### Mice and pathological studies

Mice were maintained in pathogen-free sterile isolators, according to the guidelines of the Council of Agriculture (COA, Taiwan), and all food, water, caging, and bedding were sterilized before use. The animal protocol (103034) was approved by the Institutional Animal Care and Use Committee (IACUC) of the College of Life Science, National Taiwan Ocean University. Female BALB/c nu/nu mice were obtained from BioLASCO Taiwan, Co., Ltd., and housed at the Laboratory Animal Facility, National Taiwan Ocean University, Keelung, Taiwan. For the TP4 treatment assay, nude mice with pre-growth MB231 tumors (*n* = 5 for each group) were subcutaneously injected with TP4 [500 μg in 50 μL distilled water plus 10 μL KY jelly (Johnson & Johnson)] every two days for a total of fourteen times, by which time the tumors had reached an average volume of 30-50 mm^3^ in size. Age-matched control nude mice without tumor xenografts were injected with KY jelly (10 μL plus 50 μL distilled water). Tumor size was calculated every two days, using the following formula: volume = [(height×length×width) × 3.1416]/6. Mice were sacrificed 28 days after the beginning of TP4 treatment, and the tumors were harvested and weighed. Tumor samples were fixed with formalin and embedded with paraffin. Paraffin sections were stained by Hematoxylin & Eosin (H&E) and immunostained with Ki-67 antibody (Cell Signaling, clone D2H10, 1:100). Images were obtained with an inverted microscope (Olympus, IX71) coupled to a digital camera (Olympus DP80), using a 10× (UPlanFI 10× /0.30 Ph1) and 40× (LUCPlanFI 40× /0.60 Ph2) objective lens. CellSens standard software was used for image acquisition. Fluorescent images were obtained with an FV1000 laser-scanning confocal microscope, using a 10× objective lens (UPlanSApo 10×, N.A. 0.40). ASW2.1 software was used for image acquisition and analysis.

### Zebrafish xenotransplantation model

Fish-lines were cultured at the Marine Research Station, Institute of Cellular and Organismic Biology, Academia Sinica. All fish experimental procedures were in accordance with Academia Sinica guidelines and were approved by the “Ethical Committee for using vertebrates as experimental animals”. AB line zebrafish (*Danio rerio*) were provided by the Taiwan Zebrafish Core Facility (Academia Sinica). The transgenic line (*fli:eGFP*) was purchased from JY LIN Trading Co., Ltd (Pingtung, Taiwan). Tumor cell xenotransplantation protocols were performed in accordance with previously published methods with modifications [58, 59]. Briefly, six-month-old adult AB or transgenic strain zebrafish were used for mating. Fertilized zebrafish eggs were incubated at 28°C in E3 embryo medium (5 mM NaCl, 0.17 mM KCl, 0.33 mM MgSO_4_) containing 0.2 mM PTU (Sigma). After de-chorionization at 24 hpf (hour-post-fertilization), eggs were soaked in E3 medium with tricaine (0.02 mg mL^−1^, Sigma). After 24h (48 hpf), embryos were orientated on a 1.8% agarose-modified microinjection plate. Tumor cells (2 × 10^6^ of MB231 or M10 cells expressing eGFP/mOrange2) were suspended in 25 μL Matrigel® matrix (12.0 mg mL^−1^) solution (Corning), and 10-15 nL cell suspensions were microinjected into embryos (parameters were set at 7.0 psi and 0.5-1.0 secs). Xenografted embryos were placed in a 96-well black plate with a clear bottom (Coring) and then immobilized with methyl cellulose (1.25 μL); images were obtained with an inverted microscope (Olympus IX71) equipped with a camera (Olympus DP80), using a 4× objective lens (Olympus UPlanFI 4×/0.13 phL). On every subsequent day for 5 days, the media in each well were replaced with fresh E3 media containing TP4 (3 μg mL^−1^), and images were obtained. The fluorescent signal (which is proportional to the number of eGFP-expressing cells) was quantitatively determined using Image J software. For time-lapse studies, immobilized and xenograft embryos received a single dose of TP4 or mock treatment before imaging and were incubated at 28°C for 48h. Images were obtained using the ImageXpress Micro HCS Image System (Molecular Devices). Images (including *z* stacks) were recorded under a 4× objective lens (Plan Fluor 4×/0.13,) at 1h intervals, using transmitted light and the FITC (EX 482/35, EM 536/40) and TRITC (EX 543/22, EM 593/40) filter sets. Every channel was captured from 5 images along the *z*-axis across a distance of 70 μm, and was composited to the best-focus image. Images were taken and tumor analysis was performed using the integrated MetaXpress® program (v.5.3, Custom Module Editor) to quantify the area and fluorescence intensity of the tumor inside the zebrafish. Normalized data are expressed relative to the value at 0h.

### Statistical analysis

For the multi-well based assay, cells were plated at least in sextuplicate. Data were collected from independently repeated experiments (*N* ≥ 3) and were analyzed by Prism 5 software (GraphPad Inc.). The statistical significance of any difference was determined by applying the two-tailed *t*-test or one-way/two-way analysis of variance (*ANOVA*) with Bonferroni post-test. The difference was considered statistically significant at *P* < 0.05.

## SUPPLEMENTARY FIGURES AND TABLES





## References

[1] Perou CM, Sorlie T, Eisen MB, van de Rijn M, Jeffrey SS, Rees CA, Pollack JR, Ross DT, Johnsen H, Akslen LA, Fluge O, Pergamenschikov A, Williams C, Zhu SX, Lonning PE, Borresen-Dale AL (2000). Molecular portraits of human breast tumours. Nature.

[2] Sorlie T, Perou CM, Tibshirani R, Aas T, Geisler S, Johnsen H, Hastie T, Eisen MB, van de Rijn M, Jeffrey SS, Thorsen T, Quist H, Matese JC, Brown PO, Botstein D, Lonning PE (2001). Gene expression patterns of breast carcinomas distinguish tumor subclasses with clinical implications. Proceedings of the National Academy of Sciences of the United States of America.

[3] Parker JS, Mullins M, Cheang MC, Leung S, Voduc D, Vickery T, Davies S, Fauron C, He X, Hu Z, Quackenbush JF, Stijleman IJ, Palazzo J, Marron JS, Nobel AB, Mardis E (2009). Supervised risk predictor of breast cancer based on intrinsic subtypes. Journal of clinical oncology.

[4] Rouzier R, Perou CM, Symmans WF, Ibrahim N, Cristofanilli M, Anderson K, Hess KR, Stec J, Ayers M, Wagner P, Morandi P, Fan C, Rabiul I, Ross JS, Hortobagyi GN, Pusztai L (2005). Breast cancer molecular subtypes respond differently to preoperative chemotherapy. Clinical cancer research.

[5] Carey LA, Dees EC, Sawyer L, Gatti L, Moore DT, Collichio F, Ollila DW, Sartor CI, Graham ML, Perou CM (2007). The triple negative paradox: primary tumor chemosensitivity of breast cancer subtypes. Clinical cancer research.

[6] O'Driscoll L, Clynes M (2006). Biomarkers and multiple drug resistance in breast cancer. Current cancer drug targets.

[7] Naumov GN, Townson JL, MacDonald IC, Wilson SM, Bramwell VH, Groom AC, Chambers AF (2003). Ineffectiveness of doxorubicin treatment on solitary dormant mammary carcinoma cells or late-developing metastases. Breast cancer research and treatment.

[8] O'shaughnessy J, Miles D, Vukelja S, Moiseyenko V, Ayoub JP, Cervantes G, Fumoleau P, Jones S, Lui WY, Mauriac L, Twelves C, Van Hazel G, Verma S, Leonard R (2002). Superior survival with capecitabine plus docetaxel combination therapy in anthracycline-pretreated patients with advanced breast cancer: phase III trial results. Journal of clinical oncology.

[9] Jassem J, Carroll C, Ward SE, Simpson E, Hind D (2009). The clinical efficacy of cytotoxic agents in locally advanced or metastatic breast cancer patients pretreated with an anthracycline and a taxane: a systematic review. Eur J Cancer.

[10] Jones A, O'Brien M, Sommer H, Nowara E, Welt A, Pienkowski T, Rolski J, Pham ML, Perraud K, Trillet-Lenoir V (2010). Phase II study of oral vinorelbine in combination with capecitabine as second line chemotherapy in metastatic breast cancer patients previously treated with anthracyclines and taxanes. Cancer chemotherapy and pharmacology.

[11] Stemmler HJ, diGioia D, Freier W, Tessen HW, Gitsch G, Jonat W, Brugger W, Kettner E, Abenhardt W, Tesch H, Hurtz HJ, Rosel S, Brudler O, Heinemann V (2011). Randomised phase II trial of gemcitabine plus vinorelbine vs gemcitabine plus cisplatin vs gemcitabine plus capecitabine in patients with pretreated metastatic breast cancer. British journal of cancer.

[12] Bramati A, Girelli S, Torri V, Farina G, Galfrascoli E, Piva S, Moretti A, Dazzani MC, Sburlati P, La Verde NM (2014). Efficacy of biological agents in metastatic triple-negative breast cancer. Cancer treatment reviews.

[13] Riedl S, Rinner B, Asslaber M, Schaider H, Walzer S, Novak A, Lohner K, Zweytick D (2011). In search of a novel target - phosphatidylserine exposed by non-apoptotic tumor cells and metastases of malignancies with poor treatment efficacy. Biochimica et biophysica acta.

[14] Hilchie AL, Doucette CD, Pinto DM, Patrzykat A, Douglas S, Hoskin DW (2011). Pleurocidin-family cationic antimicrobial peptides are cytolytic for breast carcinoma cells and prevent growth of tumor xenografts. Breast cancer research.

[15] Hallock KJ, Lee DK, Omnaas J, Mosberg HI, Ramamoorthy A (2002). Membrane composition determines pardaxin's mechanism of lipid bilayer disruption. Biophysical journal.

[16] Gottler LM, Ramamoorthy A (2009). Structure, membrane orientation, mechanism, and function of pexiganan--a highly potent antimicrobial peptide designed from magainin. Biochimica et biophysica acta.

[17] Ramamoorthy A, Lee DK, Narasimhaswamy T, Nanga RP (2010). Cholesterol reduces pardaxin's dynamics-a barrel-stave mechanism of membrane disruption investigated by solid-state NMR. Biochimica et biophysica acta.

[18] Zasloff M (2002). Antimicrobial peptides of multicellular organisms. Nature.

[19] Zanetti M (2004). Cathelicidins, multifunctional peptides of the innate immunity. Journal of leukocyte biology.

[20] Powers JP, Hancock RE (2003). The relationship between peptide structure and antibacterial activity. Peptides.

[21] Papo N, Shahar M, Eisenbach L, Shai Y (2003). A novel lytic peptide composed of DL-amino acids selectively kills cancer cells in culture and in mice. The Journal of biological chemistry.

[22] Hoskin DW, Ramamoorthy A (2008). Studies on anticancer activities of antimicrobial peptides. Biochimica et biophysica acta.

[23] Ting CH, Huang HN, Huang TC, Wu CJ, Chen JY (2014). The mechanisms by which pardaxin, a natural cationic antimicrobial peptide, targets the endoplasmic reticulum and induces c-FOS. Biomaterials.

[24] Papo N, Braunstein A, Eshhar Z, Shai Y (2004). Suppression of human prostate tumor growth in mice by a cytolytic D-, L-amino Acid Peptide: membrane lysis, increased necrosis, and inhibition of prostate-specific antigen secretion. Cancer research.

[25] Rodrigues EG, Dobroff AS, Cavarsan CF, Paschoalin T, Nimrichter L, Mortara RA, Santos EL, Fazio MA, Miranda A, Daffre S, Travassos LR (2008). Effective topical treatment of subcutaneous murine B16F10-Nex2 melanoma by the antimicrobial peptide gomesin. Neoplasia.

[26] Chen JY, Lin WJ, Lin TL (2009). A fish antimicrobial peptide, tilapia hepcidin TH2-3, shows potent antitumor activity against human fibrosarcoma cells. Peptides.

[27] Lin WJ, Chien YL, Pan CY, Lin TL, Chen JY, Chiu SJ, Hui CF (2009). Epinecidin-1, an antimicrobial peptide from fish (Epinephelus coioides) which has an antitumor effect like lytic peptides in human fibrosarcoma cells. Peptides.

[28] Gaspar D, Veiga AS, Sinthuvanich C, Schneider JP, Castanho MA (2012). Anticancer peptide SVS-1: efficacy precedes membrane neutralization. Biochemistry.

[29] Wang C, Li HB, Li S, Tian LL, Shang DJ (2012). Antitumor effects and cell selectivity of temporin-1CEa, an antimicrobial peptide from the skin secretions of the Chinese brown frog (Rana chensinensis). Biochimie.

[30] Kawamoto M, Horibe T, Kohno M, Kawakami K (2011). A novel transferrin receptor-targeted hybrid peptide disintegrates cancer cell membrane to induce rapid killing of cancer cells. BMC cancer.

[31] Huang TC, Chen JY (2013). Proteomic analysis reveals that pardaxin triggers apoptotic signaling pathways in human cervical carcinoma HeLa cells: cross talk among the UPR, c-Jun and ROS. Carcinogenesis.

[32] Leuschner C, Enright FM, Gawronska B, Hansel W (2003). Membrane disrupting lytic peptide conjugates destroy hormone dependent and independent breast cancer cells in vitro and in vivo. Breast cancer research and treatment.

[33] Leuschner C, Hansel W (2005). Targeting breast and prostate cancers through their hormone receptors. Biology of reproduction.

[34] van Zoggel H, Carpentier G, Dos Santos C, Hamma-Kourbali Y, Courty J, Amiche M, Delbe J (2012). Antitumor and angiostatic activities of the antimicrobial peptide dermaseptin B2. PloS one.

[35] Wang C, Tian LL, Li S, Li HB, Zhou Y, Wang H, Yang QZ, Ma LJ, Shang DJ (2013). Rapid cytotoxicity of antimicrobial peptide tempoprin-1CEa in breast cancer cells through membrane destruction and intracellular calcium mechanism. PloS one.

[36] Eferl R, Wagner EF (2003). AP-1: a double-edged sword in tumorigenesis. Nature reviews Cancer.

[37] Milde-Langosch K, Roder H, Andritzky B, Aslan B, Hemminger G, Brinkmann A, Bamberger CM, Loning T, Bamberger AM (2004). The role of the AP-1 transcription factors c-Fos, FosB, Fra-1 and Fra-2 in the invasion process of mammary carcinomas. Breast cancer research and treatment.

[38] Zhao C, Qiao Y, Jonsson P, Wang J, Xu L, Rouhi P, Sinha I, Cao Y, Williams C, Dahlman-Wright K (2014). Genome-wide profiling of AP-1-regulated transcription provides insights into the invasiveness of triple-negative breast cancer. Cancer research.

[39] Bamberger AM, Methner C, Lisboa BW, Stadtler C, Schulte HM, Loning T, Milde-Langosch K (1999). Expression pattern of the AP-1 family in breast cancer: association of fosB expression with a well-differentiated, receptor-positive tumor phenotype. International journal of cancer.

[40] Milde-Langosch K, Kappes H, Riethdorf S, Loning T, Bamberger AM (2003). FosB is highly expressed in normal mammary epithelia, but down-regulated in poorly differentiated breast carcinomas. Breast cancer research and treatment.

[41] Peng KC, Lee SH, Hour AL, Pan CY, Lee LH, Chen JY (2012). Five different piscidins from Nile tilapia, Oreochromis niloticus: analysis of their expressions and biological functions. PloS one.

[42] Morash MG, Douglas SE, Robotham A, Ridley CM, Gallant JW, Soanes KH (2011). The zebrafish embryo as a tool for screening and characterizing pleurocidin host-defense peptides as anti-cancer agents. Disease models & mechanisms.

[43] Karin M (1995). The regulation of AP-1 activity by mitogen-activated protein kinases. The Journal of biological chemistry.

[44] van der Vaart M, Spaink HP, Meijer AH (2012). Pathogen recognition and activation of the innate immune response in zebrafish. Advances in hematology.

[45] Belguise K, Kersual N, Galtier F, Chalbos D (2005). FRA-1 expression level regulates proliferation and invasiveness of breast cancer cells. Oncogene.

[46] Belguise K, Milord S, Galtier F, Moquet-Torcy G, Piechaczyk M, Chalbos D (2012). The PKCtheta pathway participates in the aberrant accumulation of Fra-1 protein in invasive ER-negative breast cancer cells. Oncogene.

[47] Desmet CJ, Gallenne T, Prieur A, Reyal F, Visser NL, Wittner BS, Smit MA, Geiger TR, Laoukili J, Iskit S, Rodenko B, Zwart W, Evers B, Horlings H, Ajouaou A, Zevenhoven J (2013). Identification of a pharmacologically tractable Fra-1/ADORA2B axis promoting breast cancer metastasis. Proceedings of the National Academy of Sciences of the United States of America.

[48] Wagner EF, Nebreda AR (2009). Signal integration by JNK and p38 MAPK pathways in cancer development. Nature reviews Cancer.

[49] Singel SM, Batten K, Cornelius C, Jia G, Fasciani G, Barron SL, Wright WE, Shay JW (2014). Receptor-interacting protein kinase 2 promotes triple-negative breast cancer cell migration and invasion via activation of nuclear factor-kappaB and c-Jun N-terminal kinase pathways. Breast cancer research.

[50] Qi X, Yin N, Ma S, Lepp A, Tang J, Jing W, Johnson B, Dwinell MB, Chitambar CR, Chen G (2015). p38gamma MAPK Is a Therapeutic Target for Triple-Negative Breast Cancer by Stimulation of Cancer Stem-Like Cell Expansion. Stem Cells.

[51] Gewirtz DA (1999). A critical evaluation of the mechanisms of action proposed for the antitumor effects of the anthracycline antibiotics adriamycin and daunorubicin. Biochemical pharmacology.

[52] Jensen PB, Sorensen BS, Sehested M, Demant EJ, Kjeldsen E, Friche E, Hansen HH (1993). Different modes of anthracycline interaction with topoisomerase II. Separate structures critical for DNA-cleavage, and for overcoming topoisomerase II-related drug resistance. Biochemical pharmacology.

[53] Pang B, Qiao X, Janssen L, Velds A, Groothuis T, Kerkhoven R, Nieuwland M, Ovaa H, Rottenberg S, van Tellingen O, Janssen J, Huijgens P, Zwart W, Neefjes J (2013). Drug-induced histone eviction from open chromatin contributes to the chemotherapeutic effects of doxorubicin. Nature communications.

[54] Kuznetsov AV, Margreiter R, Amberger A, Saks V, Grimm M (2011). Changes in mitochondrial redox state, membrane potential and calcium precede mitochondrial dysfunction in doxorubicin-induced cell death. Biochimica et biophysica acta.

[55] Wang S, Konorev EA, Kotamraju S, Joseph J, Kalivendi S, Kalyanaraman B (2004). Doxorubicin induces apoptosis in normal and tumor cells via distinctly different mechanisms. intermediacy of H(2)O(2)- and p53-dependent pathways. The Journal of biological chemistry.

[56] Melcher A, Todryk S, Hardwick N, Ford M, Jacobson M, Vile RG (1998). Tumor immunogenicity is determined by the mechanism of cell death via induction of heat shock protein expression. Nature medicine.

[57] Berge G, Eliassen LT, Camilio KA, Bartnes K, Sveinbjornsson B, Rekdal O (2010). Therapeutic vaccination against a murine lymphoma by intratumoral injection of a cationic anticancer peptide. Cancer immunology, immunotherapy.

[58] Nicoli S, Presta M (2007). The zebrafish/tumor xenograft angiogenesis assay. Nature protocols.

[59] Renaud O, Herbomel P, Kissa K (2011). Studying cell behavior in whole zebrafish embryos by confocal live imaging: application to hematopoietic stem cells. Nature protocols.

