# Supplementary material for: Targeting FOSB with a cationic antimicrobial peptide, TP4, for treatment of triple-negative breast cancer
Source: Oncotarget. 2016 May 26;7(26):40329–47. doi: 10.18632/oncotarget.9612 (PMC5130011; doi:10.18632/oncotarget.9612)
Supplement: Supplementary file 1 [file oncotarget-07-40329-s001.pdf]

SUPPLEMENTARY MATERIALS AND METHODS

Antibodies

Antibodies used in this study (for the results shown in the Supplementary Results) were as follows: β-actin (1: 5000, clone AC-15) and caspase 3 (1:1000, clone 74T2) were from ThermoFisher Scientific; Cytochrome C (1:500, clone EP1326Y) was from EMD Millipore; cleavage-Caspase 3 (1:1000, clone 5A1E), SAPK/JNK (1:1000), phospho-SAPK/JNK (1:1000, clone 81E11), ERK1/2 (1:5000), and phospho-ERK1/2 (1:5000) were from Cell signaling; P38 MAPK and phospho-P38 MAPK were from BD Transduction Laboratories.

TUNEL staining

TUNEL (TdT-mediated dUTP nick end labeling) staining was performed using the *In Situ* Cell Death Detection Kit, POD (Roche) following the standard procedures recommended by the manufacturer. Briefly, cells (MB231 and HDF) were seeded onto the chamber slide and incubated overnight. Cells were blocked, fixed, and permeabilized after TP4 treatment for 3 or 6h. The labeling solution and TUNEL reaction mixture were then added to the cells. After three washes in PBS, cells were subjected to nuclear staining by Hochest33342. Cell images were subsequently acquired using the FLoid cell imaging station (ThermoFisher Scientific). Cells treated with DNase I served as a positive control and cells untreated with terminal transferase (the enzyme mixture) served as negative control.

Quantitative real-time PCR

Zebrafish were collected (n = 10 per group, for 3 experiments, a total of 30 zebrafish) at days 1-5 and homogenized in 300 µL Qiazol (Qiagen). Homogenates were vortexed for 15 sec, left to stand at RT for 5 min, and then added to 60 µL of chloroform. The mixtures were then vortexed for 15 sec, left to stand at RT for a further 3 min, and then transferred to Phase Lock Gel™ (5 PRIME). After centrifugation at 12,000 × *g* for 15 min, the supernatants were collected and processed using the RNA extraction kit (WELGENE Biotech). For reverse transcription, 1 µg of total RNA and the ProtoScript® II First Strand cDNA Synthesis Kit (New England Biolabs) were used by following the manufacturer's recommendations. For real-time PCR, 1.5 µL cDNA and SYBR Green Real-time PCR Master Mix (TOYOBO) were used with the StepOnePlus Real-Time PCR System (Applied Biosystems, Life technologies). The PCR condition was as follows: 95°C for 1 min (holding stage); 40 cycles of 95°C for 15 sec, 60°C for 15 sec, and 72°C for 45 sec; 95°C for 15 sec, 60°C for 1 min, and 95°C for 15 sec (Melting curve stage). To analyze gene expression, the ΔΔCT method was performed with α-tubulin (*Tub-α1b*) as the calibrator gene. Primer sequences were as follows: *Tubα1b* (F:TTCCCTCTGGCTACCTATG; R:TCTTGATGGTGGCGATTGCG), *Cxcl8a* (F:CTCAC TTAGGCAAAATGACCAG; R:TTCCAATGCGTCGGC TTTC), *Ifnϕ1* (F:GCCGATACAGGATAATAACGACAG, R:AGTGTTTTGGTCCCAGTT), *Il1b* (F:TTTGTGGGAG ACAGACGGT; R:CCAACTGCTTCATTTTGTGC), *Il10* (F:AGCACTCCACAACCCCAATC; GACCCCCTTTTC CTTCATC), *Mmp9* (F:CATCCGCAACTACAAGAC; R: TCACCTGGAGGATAAGCG), *Tnfα* (F:TCTTCAA AGTCGGGTGTATG; R:GGTCATCTCTCCAGTCTAA GG), *Tnfβ* (F:GCCAAACGAAGAAGGTCAG; R:CACC GCCAACCCATTTCA).

SUPPLEMENTARY FIGURES, TABLE, MOVIES AND DATASHEET

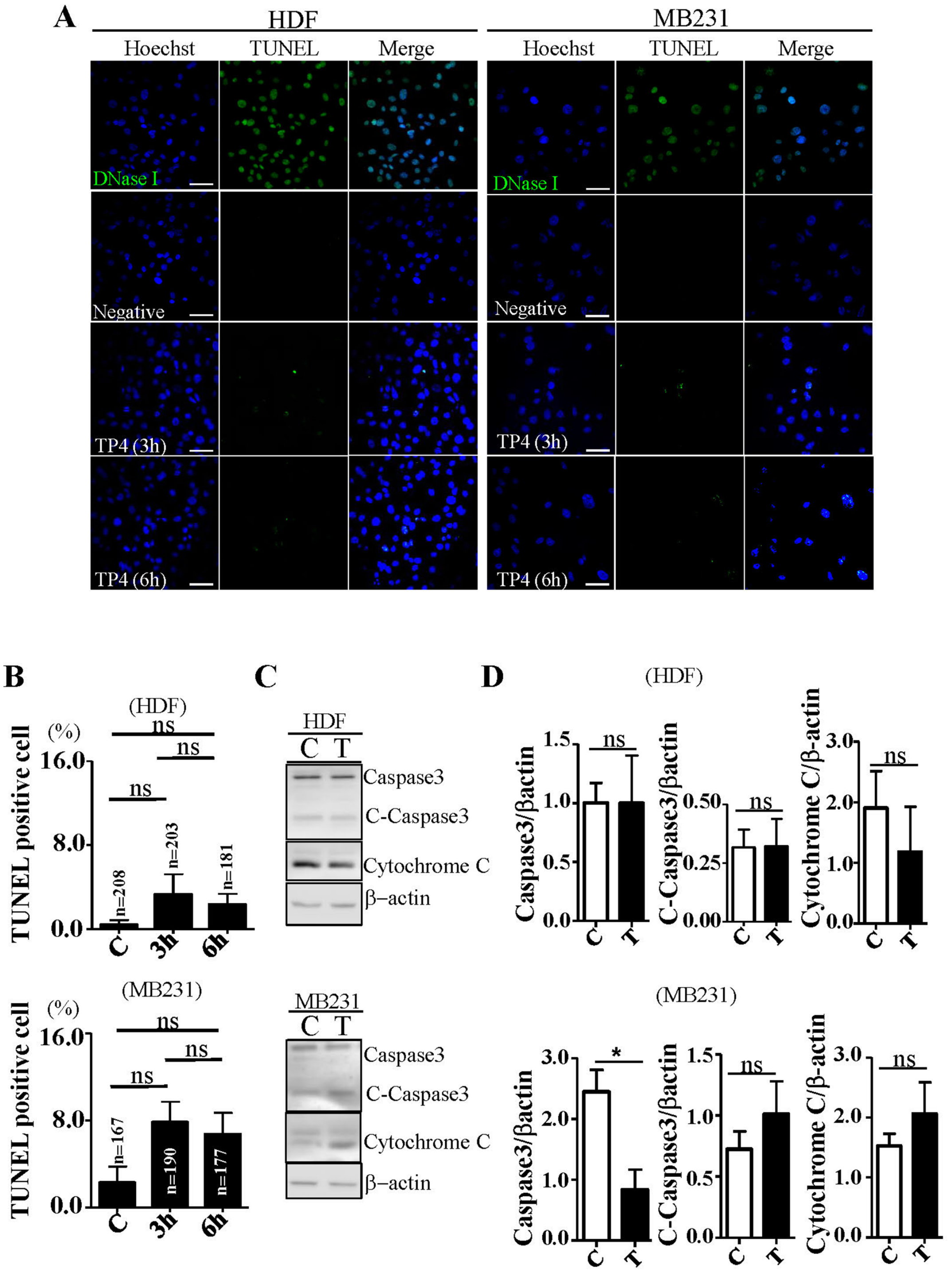

Supplementary Figure S1: TP4 does not trigger apoptosis. A. TUNEL staining was used to evaluate apoptotic cell death in HDF (left) or MB231 (right) cells treated with TP4 (14 μg mL^{-1}) for 3 or 6h. DNase I: positive control for DNA fragmentation. Bar: 50μm. **B.** Quantitation of the TUNEL-positive signals shown in (A), indicating that a very limited number of cells exhibit fluorescent labeling. Results represent the mean±SEM (Student's *t*-test: ns: not significant). **C.** Total lysates from HDF and MB231 cells incubated with (T) or without (C) TP4 were analyzed by Western blot using antibodies against Caspase3, cleaved Caspase3, Cytochrome C, and β-actin. **D.** Quantitative analyses of the blots shown in (C), using β-actin as a control for normalization. Results represent the mean±SEM from three independent experiments performed in triplicate (Student's *t*-test: *, $P < 0.05$, ns: not significant).

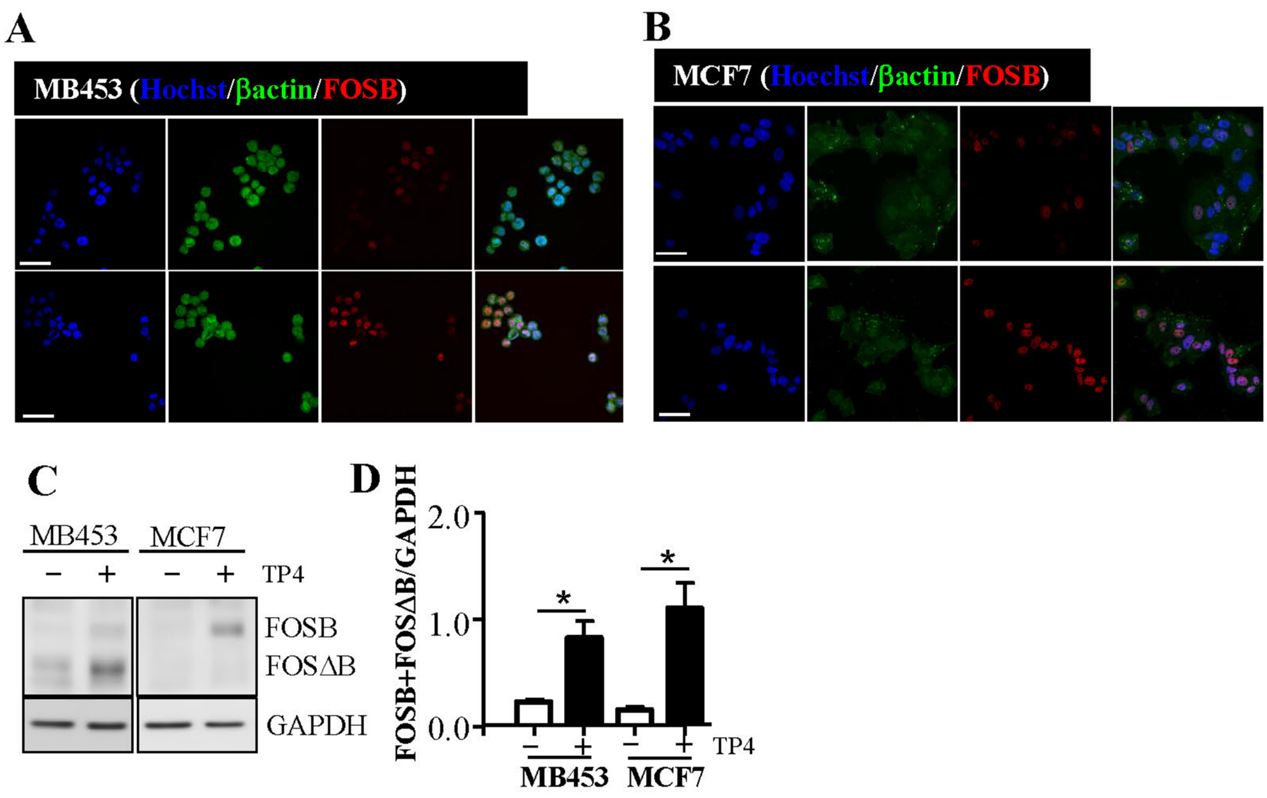

Supplementary Figure S2: FOSB is induced by TP4 in BC cells. A, B. TP4- (14 µg mL^{-1}) or mock-treated cells were stained with FOSB antibody (red) and β-actin (green). Hoechst33342 dye was used for nuclear staining (blue). Bar: 50 µm. **C.** Total lysates from MB453 and MCF7 cells treated with (+) or without (-) TP were analyzed by Western blot using antibodies against GAPDH and FOSB. **D.** Quantitative analysis of the blot shown in (C) using GAPDH as a control for normalization. Results represent the mean±SEM from three independent experiments performed in triplicate (Student's *t*-test: *, $P < 0.05$).

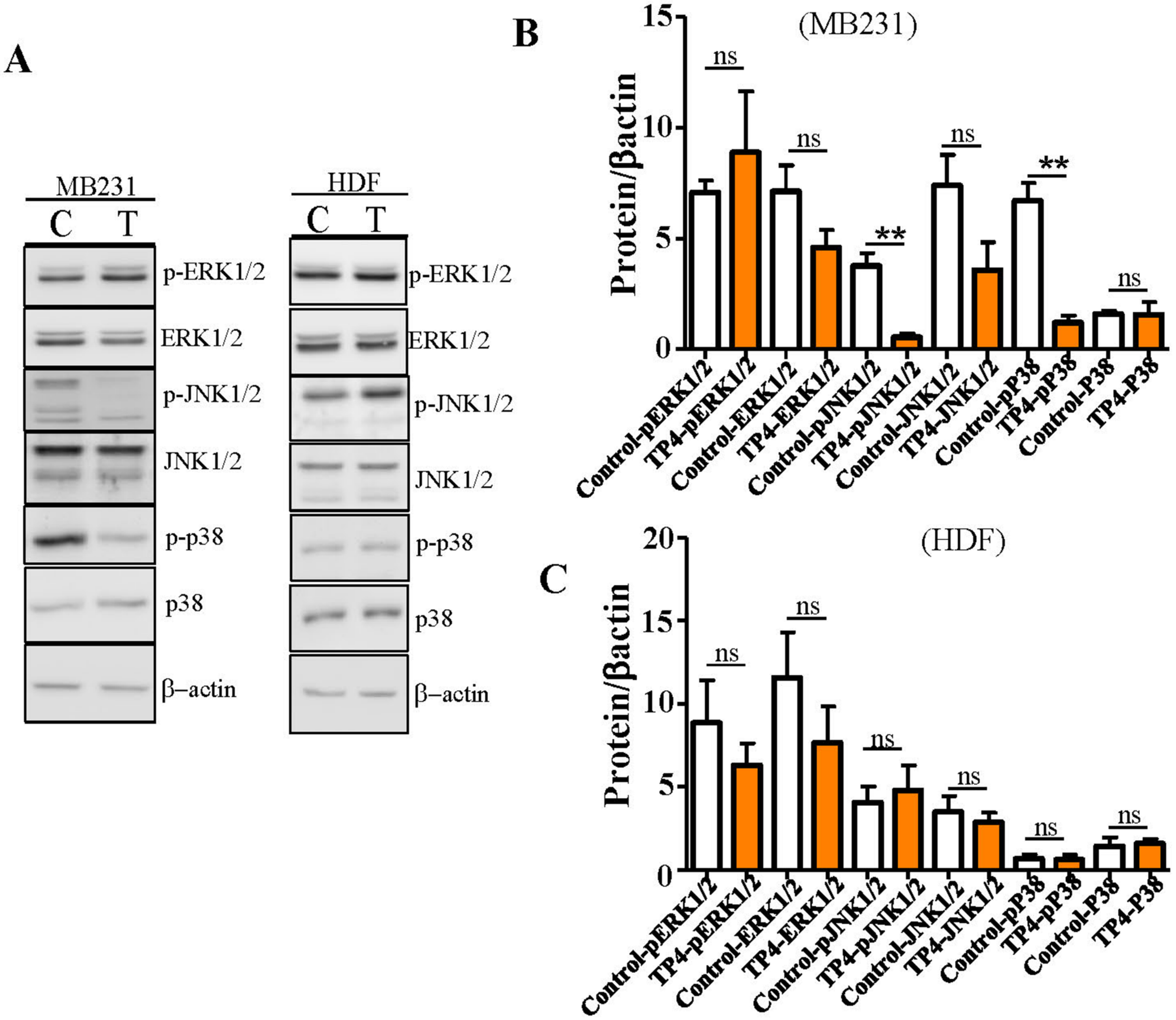

Supplementary Figure S3: MAPK pathways are affected by TP4 in MB231 cells. A. Total lysates from MB231 and HDF cells treated with (T) or without (C) TP4 were analyzed by Western blot using antibodies against ERK, phospho-ERK, JNK, phospho-JNK, p38, phospho-p38, and β-actin. **B, C.** Quantitative analyses of the blots shown in (A), using β-actin as a control for normalization. Results represent the mean±SEM from three independent experiments performed in triplicate (Student's *t*-test: **, $P < 0.01$, ns: not significant).

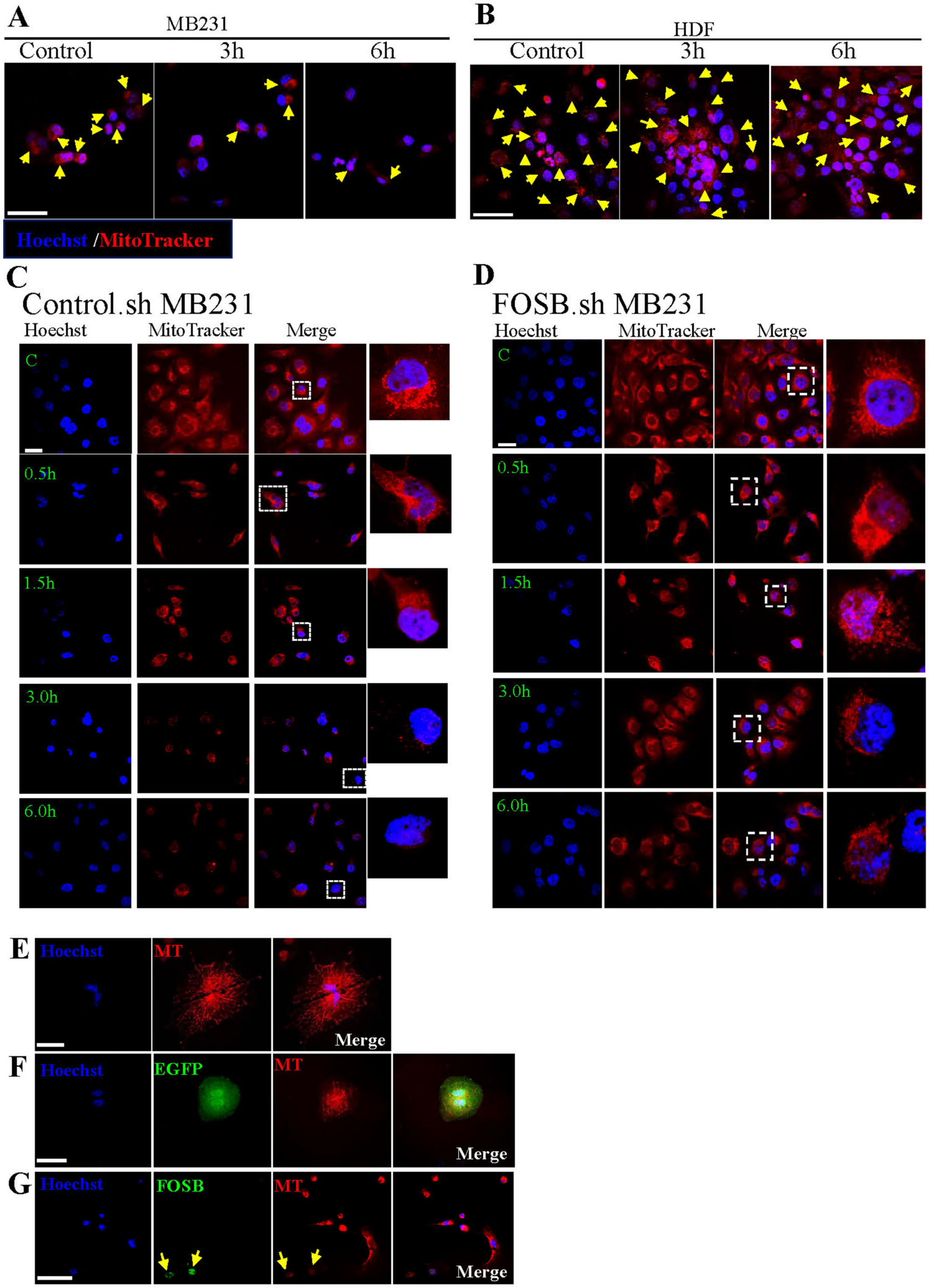

Supplementary Figure S4: Mitochondrial dysfunction caused by TP4 treatment through FOSB induction. A, B. MB231 and HDF cells were treated with TP4 (14 μg mL^{-1}) for 3 or 6h. Mitochondria were detected by staining with MitoTracker Red CMX-ROS dye. Hoechst33342 was used for nuclei staining (blue). **C, D, E.** MB231 cells were stained by MitoTracker Red CMX-ROS dye. (D) MB231 cells expressing eGFP showed similar mitochondrial staining patterns to those shown in (C). (E) MB231 cells expressing FOSB-tGFP showed a decrease of mitochondrial intensity compared with non-transfected cells. MT: MitoTracker. Scale bar: 50 μm. **F, G.** Contsh-MB231 (F) and FOSBsh-MB231 (G, indicated by arrows) cells were treated with TP4 (14 μg mL^{-1}) for 0.5-6h. Mitochondria (red) were detected by staining with MitoTracker Red CMX-ROS dye. Hoechst33342 was used for nuclei staining (blue). Boxed regions are shown magnified in the boxes to the right of the merged figures. Bar: 30 μm.

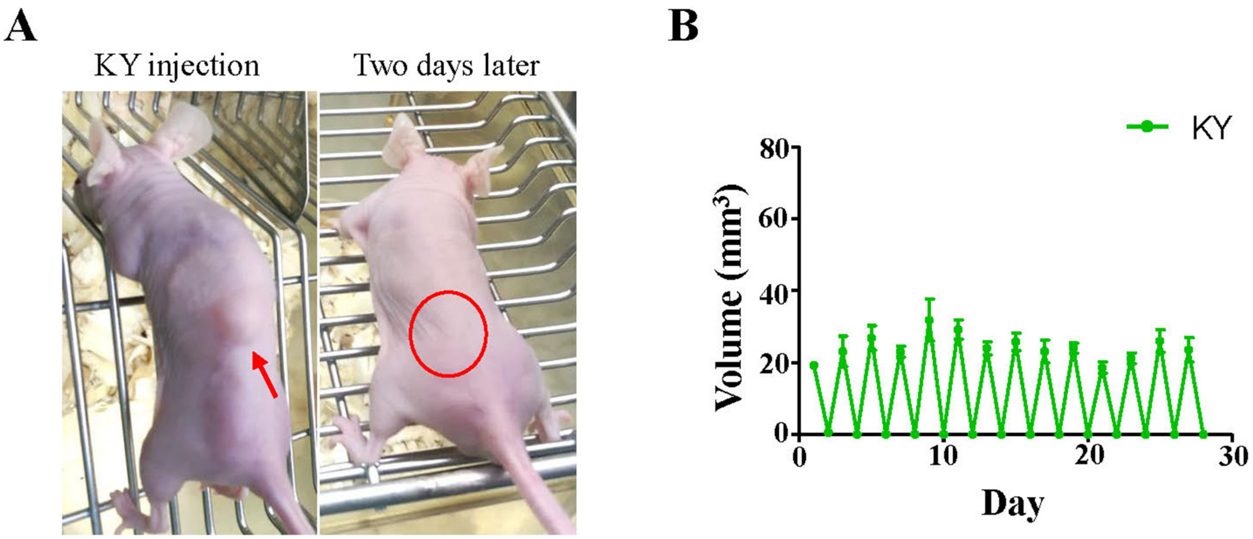

Supplementary Figure S5: KY jelly is well-absorbed in null mice. A. Nude mice (*n* = 5) were subcutaneously injected with 10 µL KY jelly plus 50 µL distilled water every two days for a total of fourteen injections. **B.** The size of the injection mixture was calculated every two days. Results represent the mean±SEM.

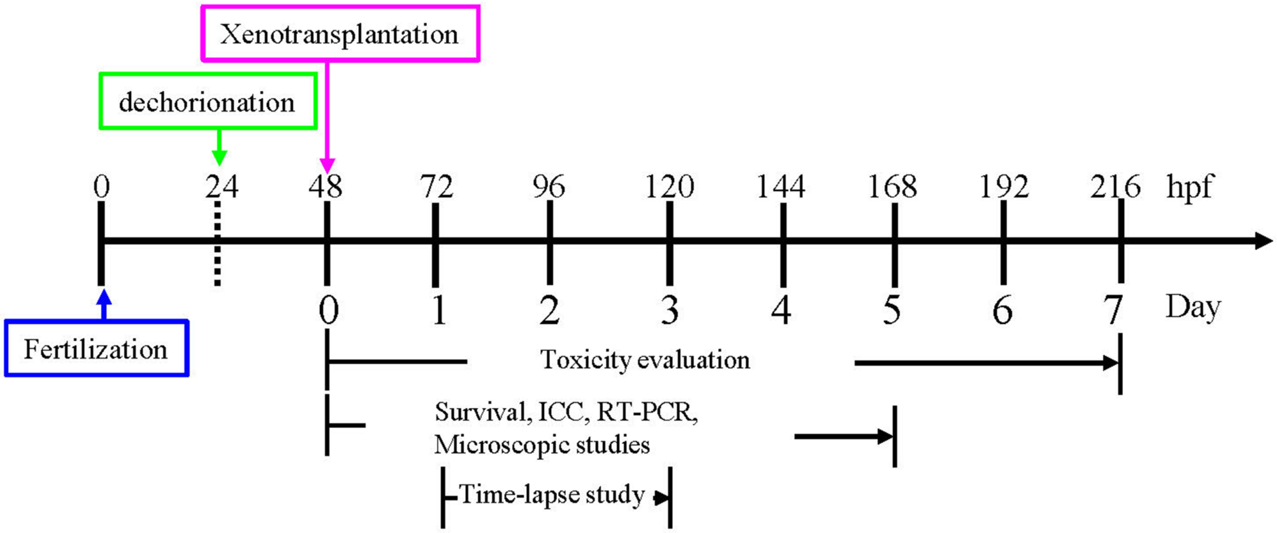

Supplementary Figure S6: A schematic diagram outlining the design of the zebrafish xenograft study. Experiments conducted in zebrafish embryos at 48 hpf are shown.

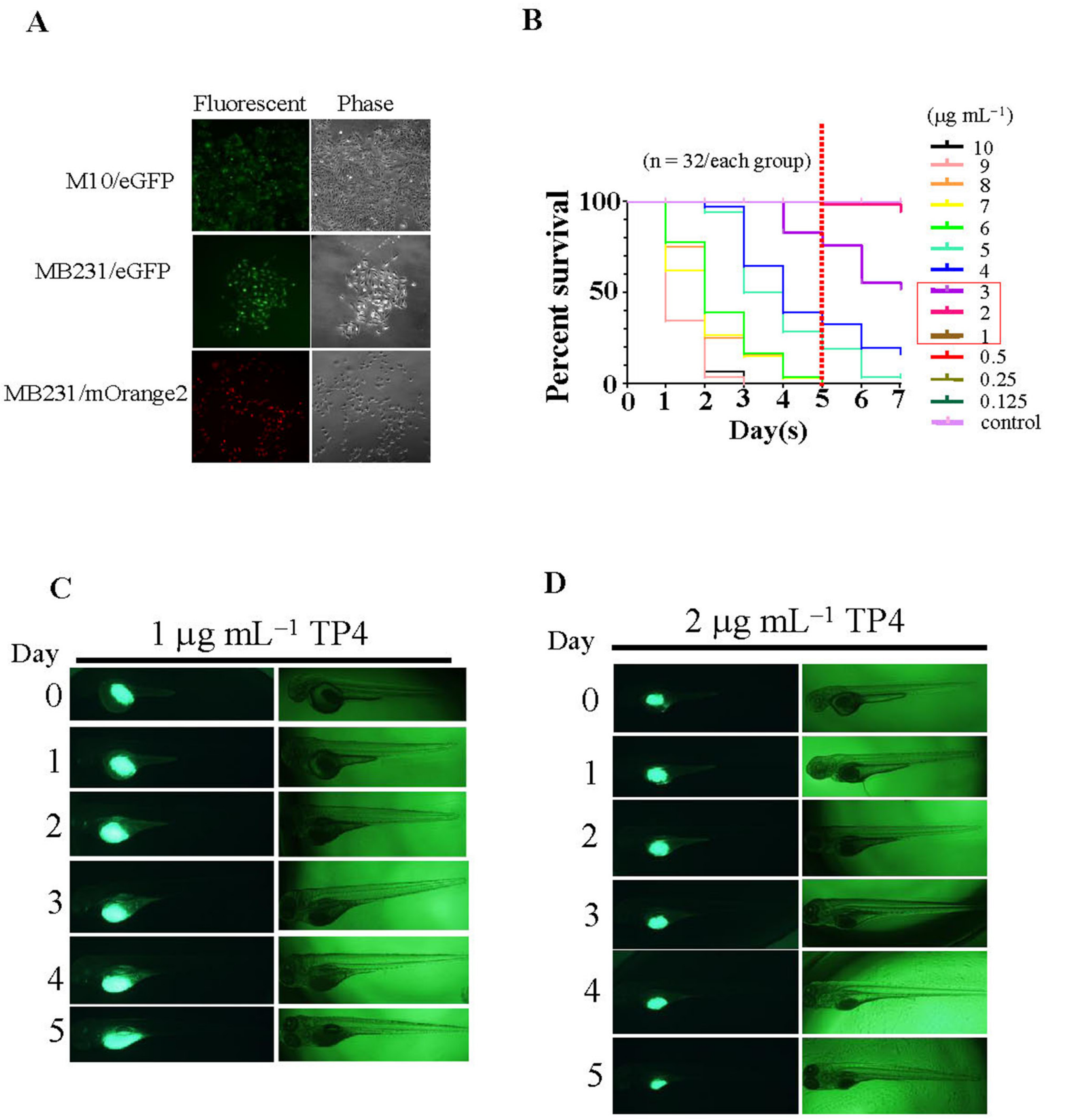

Supplementary Figure S7: Toxicity and therapeutic efficacy evaluation of TP4 in zebrafish embryos. A. Generation of fluorescent reporter cell-lines. Fluorescent stable clones (eGFP-expressing M10 or eGFP/mOrang2-expressing MB231 cells) were acquired under G418 or puromycin selection. Single clones were manipulated, expanded, and used for xenotransplantation. **B.** A series of 13 doses of TP4 were added to fish water containing zebrafish at 48 hpf every day. Survival rates were determined (n = 32 in each group). **C, D.** The eGFP fluorescent signal in TNBC xenograft zebrafish was observed under fluorescent microscopy during a five-day continuous TP4 treatment (1 or 2 μg mL^{-1}).

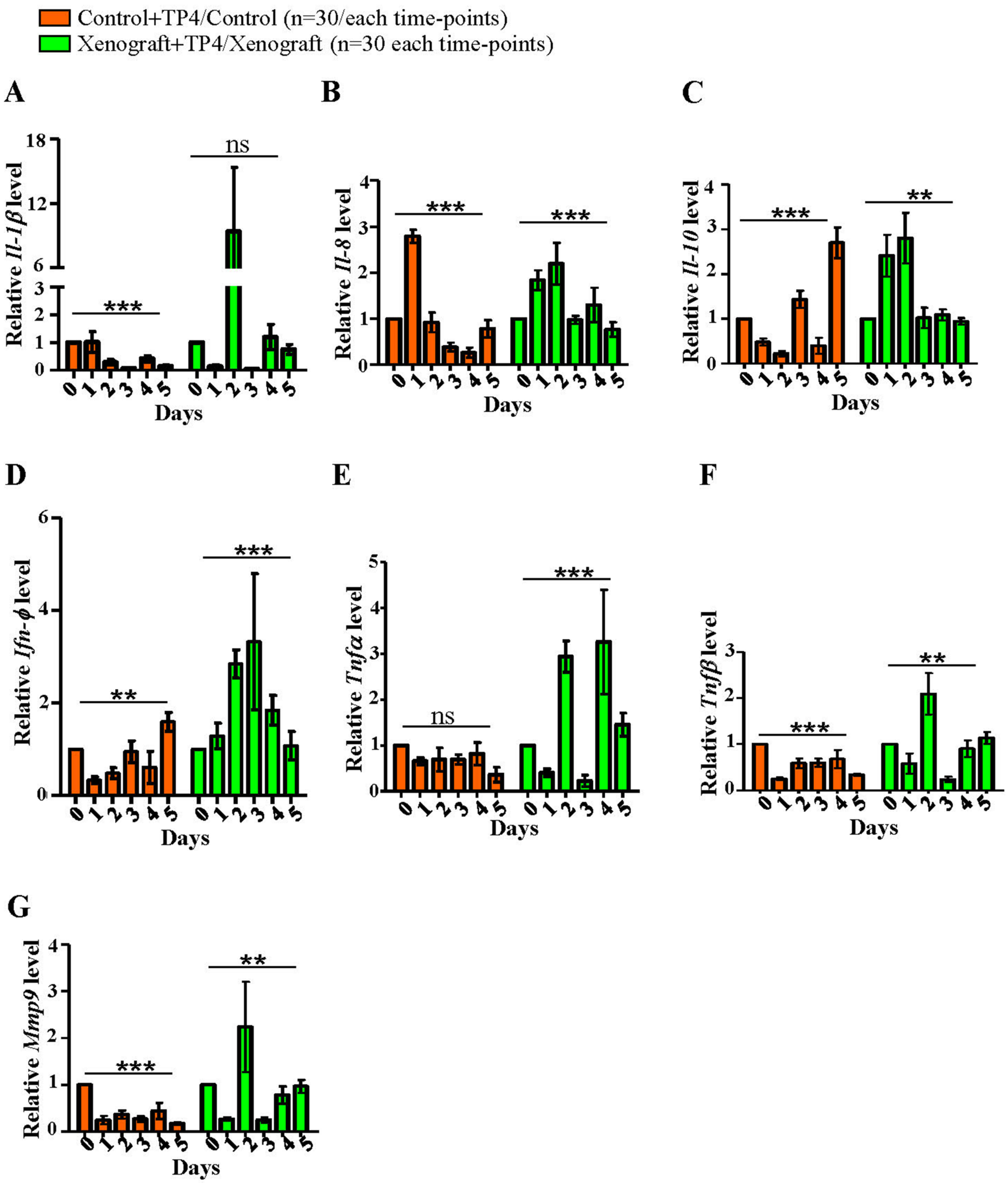

Supplementary Figure S8: TP4 enhances immunogenicity in zebrafish embryos. A–G. Real time PCR analyses of the relative immune gene expression profiles of control fish treated with or without TP4 or between TNBC xenograft fish treated with or without TP4. Statistical comparisons of immune genes between time-points were performed by one-way *ANOVA* analysis. ns: not significant; **, $P < 0.01$; ***, $P < 0.001$.

Figure 2D

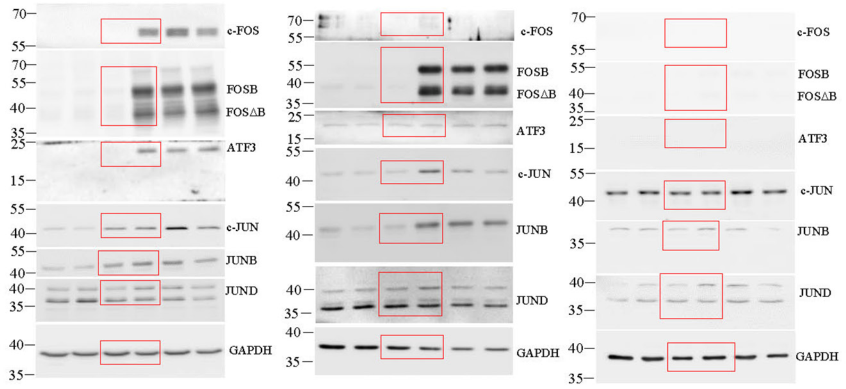

Figure 2G

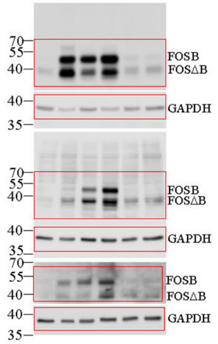

Figure 3B

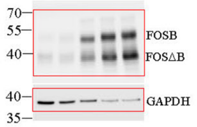

Figure 3D

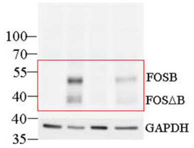

Figure 3G

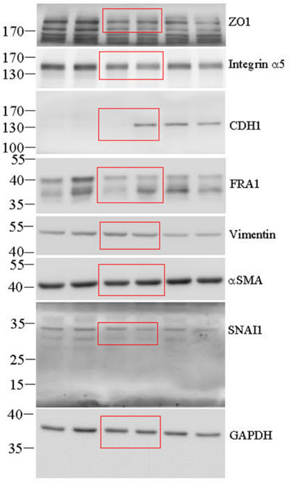

Figure 5C

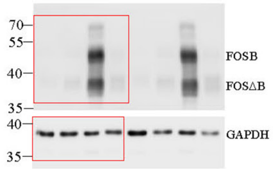

Figure 5F

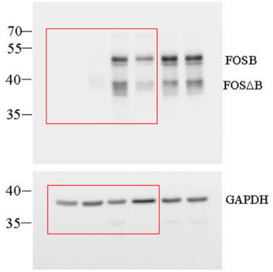

Supplementary Figure 1C

MB231

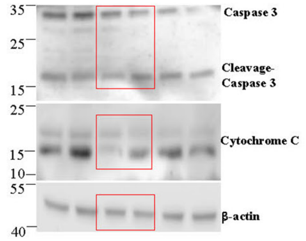

HDF

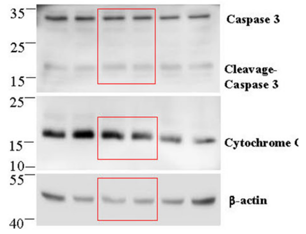

Supplementary Figure 2C

MB453

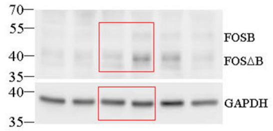

MCF7

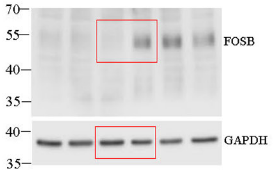

Supplementary Figure 3A

MB231

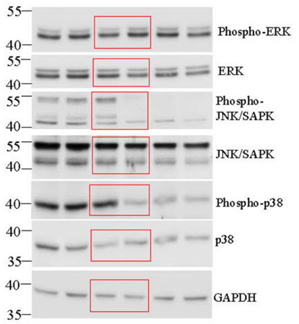

HDF

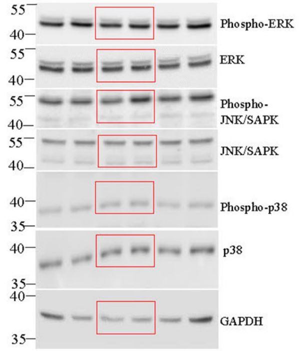

Supplementary Figure S9: Full and unedited Western blot images.

Supplementary Table S1: Cellular toxicity of TP4 to cells was evaluated by MTS assay

Time\Dose (μg/mL^{-1})	2.5	5.0	10.0	15.0	20.0
**MB231**					
3h	95.34±5.56S^{a}	90.55±10.57^{a}	75.01±11.29^{d}	52.48±11.16^{d}	30.57±4.64^{d}
6h	95.93±8.86^{a}	82.30±6.40^{d}	65.28±6.61^{d}	42.59±10.14^{d}	26.65±7.16^{d}
12h	87.60±12.28^{c}	79.00±9.48^{d}	61.63±11.38^{d}	38.31±7.26^{d}	19.31±5.73^{d}
24h	87.01±9.69^{c}	71.69±6.84^{d}	57.98±6.62^{d}	29.78±4.79^{d}	16.19±4.15^{d}
**MB453**					
3h	93.75±4.33^{d}	91.50±4.32^{d}	77.82±4.33^{d}	53.12±10.90^{d}	36.33±5067^{d}
6h	86.51±4.19^{d}	76.46±6.25^{d}	58.58±5.43^{d}	27.09±2.54^{d}	18.25±1.80^{d}
12h	92.21±6.61^{d}	80.18±5.41^{d}	62.25±10.74^{d}	27.32±5.51^{d}	15.02±4.28^{d}
24h	87.84±4.75^{d}	87.00±4.16^{d}	77.24±4.07^{d}	58.93±2.80^{d}	40.15±2.66^{d}
**MCF7**					
3h	98.00±6.05^{a}	83.74±6.10^{d}	75.01±11.15^{d}	45.15±9.89^{d}	24.63±3.80^{d}
6h	91.20±6.51^{b}	79.17±12.06^{d}	70.30±12.26^{d}	28.28±6.29^{d}	15.48±2.33^{d}
12h	93.01±10.60^{b}	77.79±7.73^{d}	68.89±6.54^{d}	26.23±6.08^{d}	14.36±2.10^{d}
24h	90.83±5.30^{b}	75.19±11.28^{d}	67.00±10.90^{d}	25.09±3.83^{d}	11.90±3.39^{d}
**M10**					
3h	100.77±4.23^{a}	97.11±4.90^{a}	90.78±4.76^{d}	84.85±5.83^{d}	67.92±5.16^{d}
6h	94.75±7.52^{c}	92.00±5.12^{d}	83.95±7.47^{d}	78.87±5.57^{d}	60.27±4.97^{d}
12h	96.05±5.66^{a}	92.62±4.99^{d}	83.77±4.27^{d}	65.49±6.68^{d}	47.16±8.42^{d}
24h	93.97±3.81^{d}	91.22±3.99^{d}	80.69±5.72^{d}	58.41±6.80^{d}	44.02±4.50^{d}
**HDF**					
3h	105.05±6.49^{a}	103.37±5.88^{a}	101.86±9.49^{1}	102.67±9027^{a}	98.64±2.96^{a}
6h	98.59±4.42^{a}	96.79±3.43^{a}	92.87±2.98^{c}	90.85±6.82^{d}	80.98±2.27^{d}
12h	100.01±4.74^{a}	93.79±3.81^{b}	89.56±3.87^{d}	85.92±4.24^{d}	80.46±1.58^{d}
24h	97.20±5.62^{a}	92.72±6.58^{c}	86.10±9.39^{d}	82.22±11.37^{d}	72.18±3.50^{d}

Statistical results from Figure 1A-1E are shown. Sextuplicate wells were analyzed for each experiment (n = 18 per dose). Results represent the mean±SD from three independent experiments. Statistical comparisons between mock versus TP4 treatment groups were performed using Two-way *ANOVA* analysis with Bonferroni post-hoc test: a, not significant; b, $P < 0.05$; c, $P < 0.01$; d, $P < 0.001$.

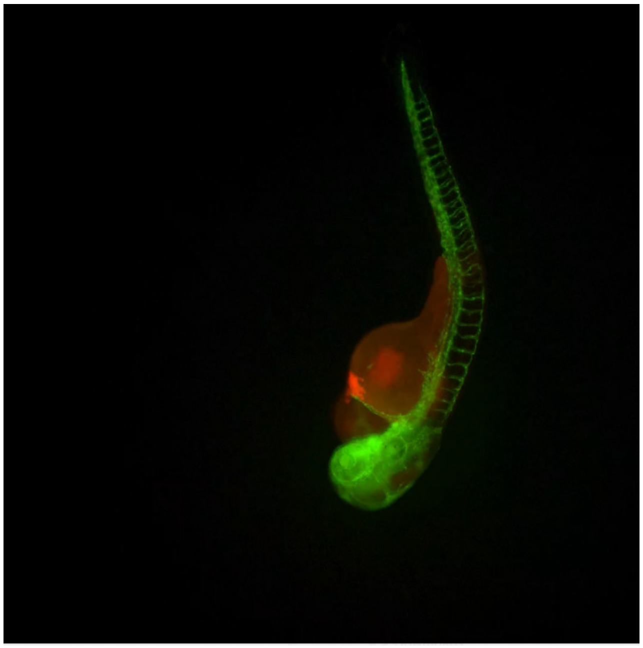

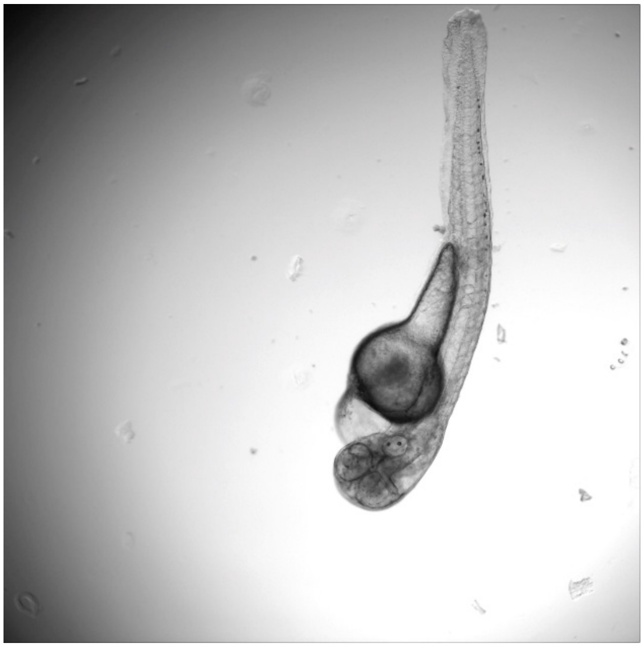

Supplementary Movies S1, S2: Xenograft zebrafish death was triggered by TNBC cell invasion. Time series images were taken every 1 h with *z*-stacks ($z = 5$). Images were constructed and shown as 6 planes/sec.

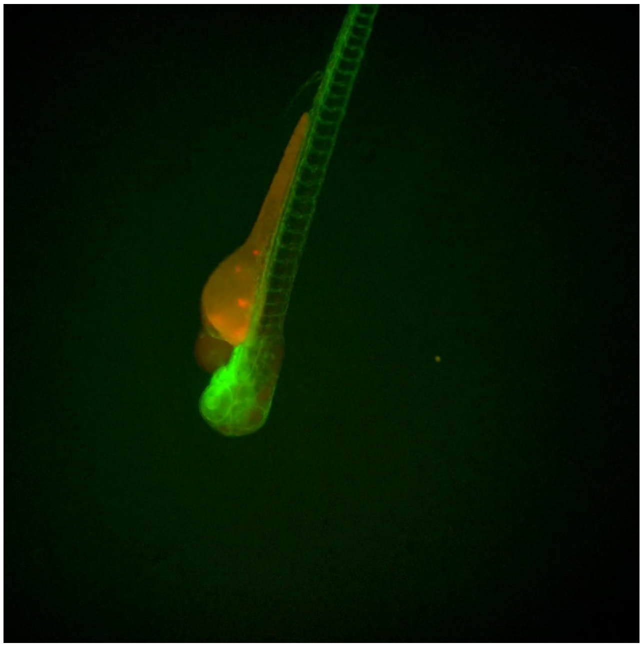

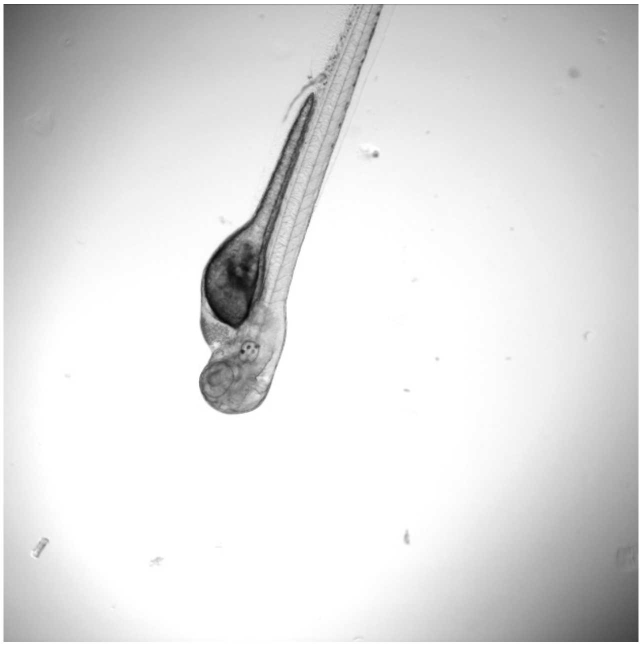

Supplementary Movies S3, S4: TP4 eliminates TNBC cells in xenograft zebrafish. Time series images were taken every 1h with *z*-stacks (*z* = 5). Images were constructed and shown as 6 planes/sec.

Supplementary Datasheet S1: Differentially-regulated genes in TP4-treated MB231 and HDF cells.

See Supplementary File 1
